# Joint associations of baseline heavy physical work and waist-to-height ratio with hospital-recorded intervertebral disc disorders: a UK Biobank cohort study

**DOI:** 10.3389/fpubh.2026.2002097

**Published:** 2026-10-28

**Authors:** Zhenhui Wang, Zhaoqing Ji, Simin Yang, Shuizhong Cen, Zhengbing Yuan

**Affiliations:** 1 Department of Spinal Surgery, Orthopedic Medical Center, Zhujiang Hospital, Southern Medical University, Guangzhou, China; 2 Neurosurgery Center, National Key Clinical Specialty, Engineering Research Center of Diagnostic and Therapeutic Technology and Devices for Cerebrovascular Diseases in Ministry of Education, Guangdong Provincial Key Laboratory on Brain Function Repair and Regeneration, Zhujiang Hospital Institute of Neurosurgery, Zhujiang Hospital, Southern Medical University, Guangzhou, China; 3 Department of Orthopedic Surgery, Dongguan Qiaotou Hospital, Dongguan, China

**Keywords:** cohort study, competing risks, hospital records, intervertebral disc disorders, occupational physical workload, radiculopathy, waist-to-height ratio

## Abstract

**Background:**

Occupational heavy physical work and central adiposity are associated with disc-related morbidity, but their joint associations and absolute risks require clarification.

**Methods:**

We studied UK Biobank participants employed or self-employed at baseline (2006–2010), without known disc disease, using linked hospital and mortality records in England, Scotland and Wales through December 31, 2015. Of 276,917 eligible participants, 270,971 with complete adjustment data formed the primary analysis population. Exposures were baseline heavy-work frequency and measured waist-to-height ratio (WHtR). Cox models evaluated continuous WHtR per 0.05 increase and four categories combining lower/higher work frequency with WHtR <0.5/≥0.5. The primary outcome was first hospital inpatient-recorded intervertebral disc disorders [International Classification of Diseases, Tenth Revision (ICD-10) M51]. The M51.1 branch in the UK Biobank coding hierarchy was a secondary, nested radiculopathy subtype. Five-year risks accounted for competing mortality.

**Results:**

Primary analyses included 2,072 disc-disorder and 951 radiculopathy-subtype events. Each 0.05 higher WHtR was associated with a disc-disorder hazard ratio (HR) of 1.13 (95% confidence interval [CI] 1.10–1.16); higher versus lower work frequency in the same model had HR 1.47 (1.31–1.65). Spline analyses provided no clear evidence of nonlinearity. Higher work frequency with WHtR ≥0.5, compared with lower frequency and WHtR <0.5, had HR 2.04 (1.75–2.38). Corresponding standardized five-year risks were 0.791% versus 0.389%, a difference of 4.03 (3.01–5.04) per 1,000 people. For the nested subtype, the continuous WHtR HR was 1.14 (1.09–1.19) and the joint-category HR was 2.00 (1.59–2.50), with a risk difference of 1.73 (1.07–2.40) per 1,000. There was no clear evidence of multiplicative or additive interaction.

**Conclusion:**

Higher baseline WHtR and more frequent heavy work were associated with subsequent hospital-recorded disc disorders. Continuous exposure estimates and modest absolute differences characterize these associations in employed adults. Repeated, task-specific measurements are needed before translating them into preventive or screening strategies.

## 1 Introduction

Intervertebral disc disorders are an important occupational-health concern for employed adults, linking back and leg symptoms with impaired work participation and specialist care (1, 2). Global burden estimates establish the scale of low back pain (3), while occupational studies provide the task-specific context needed for prevention. The challenge is to relate measurable job demands to clearly defined spinal outcomes in working populations.

Systematic reviews and primary studies have related occupational loading to radicular syndromes, hospital-treated sciatica and disc disease (4–8). Higher body mass index has also been associated with sciatica and imaging-defined disc degeneration (9, 10), and previous work examined body mass index jointly with whole-body vibration (6). These findings motivate examining occupational and anthropometric exposures together. Waist-to-height ratio (WHtR) offers a measured index of waist size relative to stature, although its cardiometabolic screening literature does not establish a disc-disease threshold (11, 12).

We examined baseline heavy-work frequency and WHtR in relation to incident hospital-recorded intervertebral disc disorders in employed UK Biobank participants. Continuous and spline analyses characterized the WHtR association, while the original four categories summarized its joint pattern with work frequency. We estimated relative hazards and standardized five-year cumulative incidence with competing death, and assessed multiplicative and additive interaction. The radiculopathy branch was analyzed as a secondary, nested clinical coding subtype. This approach quantifies a defined hospital endpoint on both relative and absolute-risk scales.

## 2 Materials and methods

### 2.1 Study design and population

UK Biobank is a prospective resource that recruited approximately half a million adults, principally aged 40–69 years, during 2006–2010 and collected baseline questionnaire, physical-measurement, and linked health-record data (13, 14). Participation was selective, so this cohort does not represent a probability sample of the UK workforce (15). The present study used baseline assessment and linked hospital and mortality records in a historical data extract. Reporting was organized with reference to STROBE and the RECORD extension for routinely collected health data (16, 17).

Participants were eligible if they were in paid employment or self-employment at baseline and had valid workload and anthropometric data. An available valid corrected employment response took precedence over the original response; nonresponse was not classified as low workload. We excluded known disc disease at or before baseline, including cervical or other intervertebral disc diagnoses, disc-specific self-reported disease or surgery, and unresolved historical or follow-up information preventing a defensible risk-time definition. Selection proceeded from 502,507 records to 276,917 eligible participants; 270,971 had complete adjustment data (Figure 1). Sources and date rules are specified in Supplementary Methods A; diagnostic and surgical exclusions in Supplementary Methods B; and employment routing in Supplementary Methods C. Study size was determined by the available eligible cohort. Precision is presented through event counts and confidence intervals rather than an effect-based retrospective power calculation.

**Figure 1:**
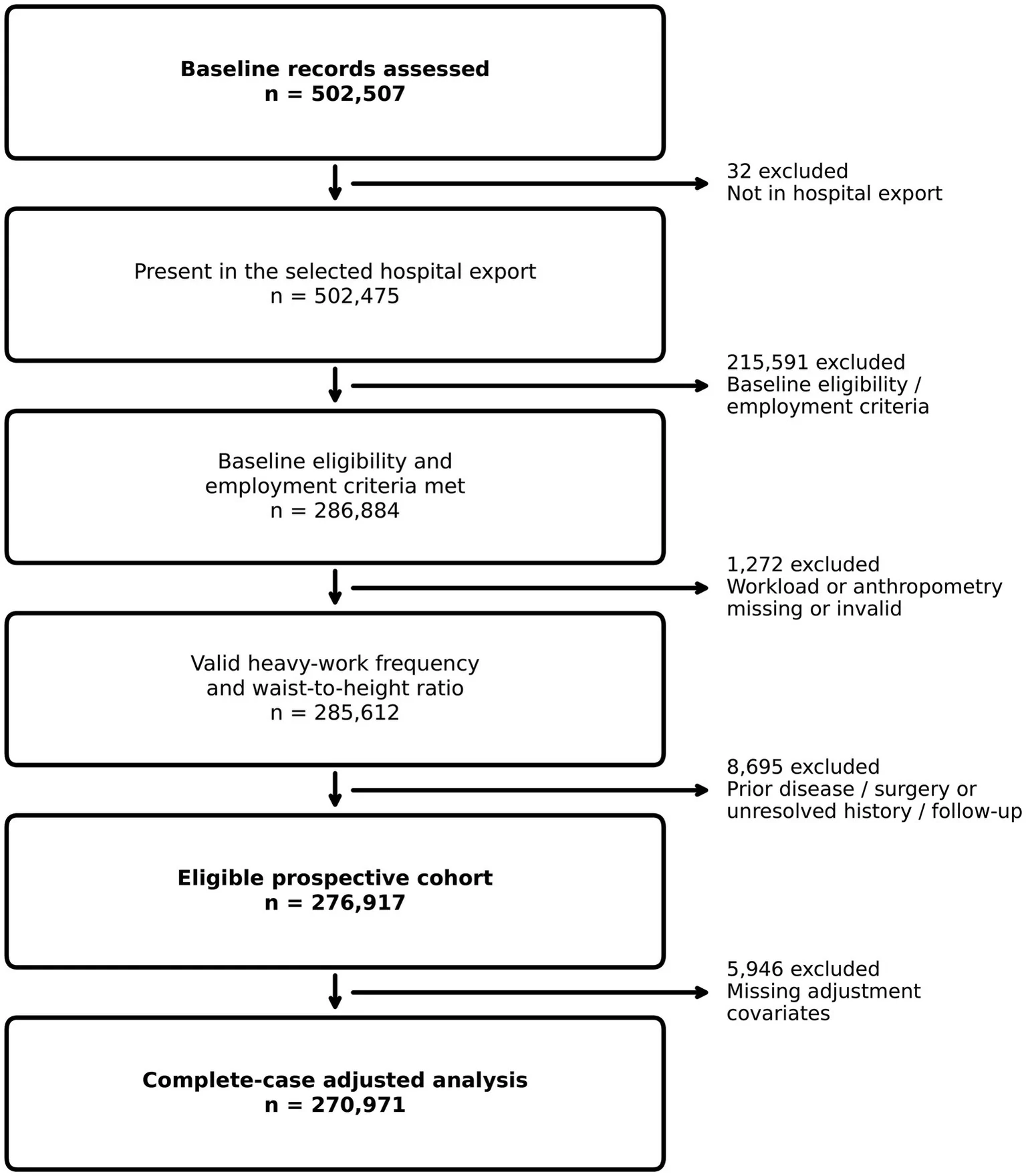
Participant selection. Eligibility was assessed at the baseline visit. The combined 8,695-person exclusion step comprises prior disc disease or disc-specific surgery and unresolved historical or follow-up information; finer components are combined for disclosure protection. The complete-case population is the target population for the fully adjusted relative-hazard and standardized 5-year risk analyses. Radiculopathy and overall disc disorders have separate follow-up variables within this population.

### 2.2 Occupational workload and central adiposity

The baseline item asked whether the current main job involved heavy physical work, with responses never/rarely, sometimes, usually, or always. Lower frequency combined never/rarely and sometimes; higher frequency combined usually and always. This grouping distinguished occasional from routine heavy work and provided a parsimonious joint-category display. It was not a biomechanical-dose boundary, and sometimes was not treated as unexposed. The original four responses were also analyzed separately. Do not know, prefer not to answer and uninterpretable responses were treated as missing. The measure concerns reported frequency at baseline, not lifting weight, posture, vibration, cumulative exposure or sustained heavy work throughout follow-up. Exact response coding is provided in Supplementary Methods C.

WHtR was calculated from measured waist circumference and standing height in the same units at baseline. Measurements had to be finite and positive; values were not winsorized. The 0.5 classification, informed by general screening literature (12), defined four groups with work frequency: lower/<0.5 (reference), higher/<0.5, lower/≥0.5 and higher/≥0.5. It was not selected to maximize an association and was not regarded as a disease-specific diagnostic or treatment boundary. Continuous WHtR and restricted cubic splines were used to characterize the association beyond this classification.

### 2.3 Outcomes and follow-up

The primary outcome was the first post-baseline hospital inpatient-recorded diagnosis in the ICD-10 M51 branch, including its subordinate codes in the UK Biobank coding hierarchy. The secondary outcome was the first record within the M51.1 branch: M511, M5116 or M5119 in the decimal-free export. We refer to this as the radiculopathy subtype. “Subordinate codes” denotes hierarchy membership, not diagnostic validation. These codes do not establish imaging-confirmed compression, electrophysiological injury, symptom severity or a purely lumbar distribution. General back pain, unlinked sciatica, spondylosis and spinal stenosis codes were not added. Exact branches and subcodes are listed in Supplementary Methods B.

UK Biobank supplied participant-linked hospital and mortality records. The hospital summary derives from admitted-patient records, including inpatient and day-case care, rather than a systematic collection of outpatient consultations. Diagnoses in any position (field 41270) were paired with first-recorded dates (41280) by matching array indices; primary-diagnosis sensitivity analyses used fields 41202 and 41262. This pairing is distinct from UK Biobank’s underlying registry linkage. An earlier non-radiculopathy M51 record neither supplied the radiculopathy date nor ended its follow-up. Consequently, the subtype has its own first-event timing but shares events and participants with the primary outcome; agreement between their estimates is not independent corroboration.

Follow-up began at baseline and ended at the corresponding outcome, death, valid recorded loss to follow-up or December 31, 2015. We used the hospital export dated May 8, 2021 and a common historical window informed by release chronology, including a subsequently documented gap in Welsh diagnosis data from 2016 onward (Supplementary Methods A). The notification concerns missing diagnosis data and does not establish that every Welsh record in that period was absent. We did not replace this extract with a newer release. Death records followed the source-conflict rules in Table M1. An outcome on the date of death, loss or administrative censoring took precedence. Death ended cause-specific risk time and was a competing event in cumulative-incidence analyses. Baseline assessment-center country defined regional strata; it was not a complete record of residence or healthcare-provider geography.

### 2.4 Covariates and model specification

Adjustment included age, sex, ethnicity, education, Townsend deprivation index, smoking and alcohol-intake frequency. These variables address demographic, socioeconomic and behavioral differences associated with occupational participation, body size and recorded morbidity. Region-specific baseline hazards address broad differences in recording and healthcare setting. This rationale does not imply that all confounding is measured. Age used a three-degree-of-freedom natural cubic spline with a fixed centered basis. Education used six nominal categories from all baseline responses: degree, A/AS levels, vocational/professional, GCSE/O levels, CSE and none of the listed qualifications; none was an observed category. Smoking was never, former or current; alcohol intake used six frequency categories. Negative Townsend scores were valid. Field definitions and the adjustment rationale are provided in Supplementary Methods C.

An initial six-category ethnicity model showed unstable estimation in the Chinese category. The authors had viewed preliminary coefficients, confidence intervals, *p* values and diagnostics when deciding to combine Chinese and Asian or Asian British responses. White, Mixed, Black or Black British and Other were retained, without removing participants. The reason was sparse-category numerical instability, not a claim of biological equivalence. Both the original model and a model stratified by the original six categories are reported alongside the pooled-category model (Supplementary Table S15). The stratified sensitivity specification was also developed after preliminary results had been viewed. Body mass index was not included in primary adjustment because the analysis did not target a WHtR association independent of overall body size. Leisure-time activity, job-specific lifting, posture and vibration were not included in the analytic adjustment set. Weekly work hours were examined in an additional analysis described below.

### 2.5 Relative hazards and model diagnostics

Cause-specific Cox proportional-hazards models estimated hazard ratios (HRs) with 95% confidence intervals (CIs) (18). Model 1 adjusted for age and sex, with regional strata. Model 2 additionally included ethnicity, education, deprivation, smoking, and alcohol intake. Model 1 was estimated in the full eligible population and again in the same complete-case population used by Model 2, separating changes due to adjustment from changes in the analytic sample. The primary contrast compared higher workload/WHtR ≥0.5 with the reference group; all other group contrasts were retained irrespective of statistical significance.

Continuous WHtR was modeled per 0.05 increase and with a four-knot restricted cubic spline at the 5th, 35th, 65th, and 95th percentiles. Spline contrasts used WHtR 0.5 as reference, with graphical reporting restricted to the 1st–99th percentile range. Such models evaluate the exposure shape without outcome-driven cut-point selection (19). These models adjusted for workload and the Model 2 covariates. Original workload frequency was modeled categorically, adjusting for continuous WHtR and the same covariates; an ordinal trend was supplementary and did not imply equal physical doses between responses.

Cox models used Efron handling of tied times. Factor-level and global Schoenfeld-residual tests evaluated proportional hazards (20). Diagnostic results are in Supplementary Table S8. To assess the influence of the nominal education and sex departures, additional models stratified baseline hazards jointly by region and education for M51, or region and sex for the radiculopathy subtype, removing the corresponding fixed-effect terms while retaining the remaining adjustment variables.

### 2.6 Five-year absolute risks and interaction

Unadjusted cumulative incidence was estimated with the Aalen–Johansen estimator. Standardized 5-year risks were derived from separate cause-specific models for the disease outcome and for death before that outcome, integrating both causes into the disease cumulative incidence (21, 22). Each of the four exposure categories was evaluated over the same empirical covariate distribution of all 270,971 complete cases. Thus, the standardized contrasts are model-based comparisons in a common target population rather than crude event proportions. Confidence intervals used the full influence-function covariance, incorporating fitted-model uncertainty and the empirical target distribution. Ten-year risks were not extrapolated beyond the supported follow-up.

We calculated each nonreference group’s risk difference from the reference group and expressed it per 1,000 people over five years. Multiplicative interaction was evaluated through the contrast β11 − β10 − β01 in the four-group Cox model. Additive interaction was evaluated directly on the standardized cumulative-incidence scale as F11(5) − F10(5) − F01(5) + F00(5), using the full four-group covariance matrix. These separate scales answer different questions (23). Interaction analyses were secondary, and a higher HR in the joint-high group alone was not interpreted as synergy.

### 2.7 Sensitivity analyses and missing data

Sensitivity analyses restricted outcomes to primary diagnoses, introduced two-year and five-year delayed entry, restricted to participants without baseline back pain, restricted body mass index to <35 kg/m^2^, and used the original ethnicity categories as strata. Within each delayed-entry analysis, all four exposure groups used the same inclusion rule: alive, free of that outcome and still observed at the relevant baseline anniversary. Start–stop risk time remained measured from baseline. No-baseline-back-pain status required interpretable pain-location responses without back pain or pain throughout the body; unknown, contradictory or inappropriately skipped responses were not negative. Rules are specified in Supplementary Methods B,C and results in Supplementary Tables S3A, S3B.

Complete-case analysis remained primary, with multiple imputation by chained equations as a sensitivity analysis under a missing-at-random assumption. We used 20 completed datasets, each generated by a separate 20-iteration chain. Only missing ethnicity, education, smoking, alcohol and Townsend values were imputed; observed values, exposures and outcome information were retained. Categorical targets used multinomial models and Townsend used predictive mean matching. Final saved predictor matrices retained exposure and covariate terms, the M51 and M51.1 event indicators, their respective competing-death indicators, and four scaled Nelson–Aalen-type cumulative-hazard auxiliaries (24–26). Estimates were combined with Rubin variance and Barnard–Rubin degrees of freedom. Fractions of missing information (FMI), final predictor inclusion and observed/completed distributions are reported in Supplementary Methods F and Supplementary Tables S13–S14. These descriptions do not establish a missing-at-random mechanism (27).

Analytic choices and later amendments were recorded; the study was not prospectively registered. Reviewer-requested analyses were additional to the original analysis plan. Two-sided *p* values were reported without selecting analyses by significance, and no omnibus multiplicity adjustment was applied. R 4.5.3 was used with survival 3.8–6, riskRegression 2026.03.11 and mice 3.19.0. Definitions, exact spline bases, imputation settings and diagnostic information are in Supplementary Methods A–G. Executed reviewer-requested model and imputation-inspection code is supplied as Supplementary Code 1.

### 2.8 Additional occupational and regional analyses

At peer review, we added analyses restricted to assessment centers in England/Scotland at the same 2015 cutoff, and models adjusting for typical weekly main-job hours (field 767, excluding commuting). Valid hours were modeled as one linear term per 10 h. Each hours-adjusted model was compared with the primary specification in the identical 267,227-person subset. These are sensitivity analyses, not a recalibration of the baseline heavy-work item. No sufficiently documented longer common observation window was available for this extract, so no new cutoff was selected. Supplementary Methods G and Supplementary Table S12 provide the specifications and complete exposure contrasts.

Repeated employment and workload responses were described only when their assessment date fell after baseline and within death/loss/administrative follow-up. Disease-free status was not required for this descriptive inventory. Repeated visits could overlap and could follow a disease event; they were not used to impute whole-cohort work histories, retirement dates or inverse-probability weights. Availability and transitions are reported in Supplementary Table S16.

## 3 Results

### 3.1 Study population and event accrual

Of 276,917 eligible participants, 270,971 (97.85%) had complete adjustment data. The reference, higher-frequency/<0.5, lower-frequency/≥0.5 and higher-frequency/≥0.5 groups contained 87,376, 10,666, 147,451 and 25,478 complete cases, respectively. The complete-case population included 141,310 women (52.1%), 94.3% White participants and 11,505 participants assessed in Wales (4.25%). Baseline characteristics are in Table 1 and Supplementary Tables S2A, S2B. Supplementary Table S10 compares complete and incomplete adjustment-data groups; Supplementary Table S13 provides missingness by exposure group and observed/completed distributions.

**Table 1:** Baseline characteristics of the complete-case population.

Characteristic	Overall *n* = 270,971	Lower workload WHtR <0.5 *n* = 87,376	Higher workload WHtR <0.5 *n* = 10,666	Lower workload WHtR ≥0.5 *n* = 147,451	Higher workload WHtR ≥0.5 *n* = 25,478
Age, years	52.7 (7.1)	51.3 (6.8)	51.1 (7.0)	53.6 (7.1)	53.1 (7.1)
Women, *n* (%)	141,310 (52.1)	63,063 (72.2)	6,089 (57.1)	64,423 (43.7)	7,735 (30.4)
WHtR	0.529 (0.073)	0.456 (0.030)	0.459 (0.029)	0.569 (0.056)	0.572 (0.056)
Townsend deprivation index	-1.35 (3.00)	-1.59 (2.89)	-0.90 (3.15)	-1.38 (2.99)	-0.51 (3.22)
White ethnicity, *n* (%)	255,422 (94.3)	83,562 (95.6)	10,039 (94.1)	138,213 (93.7)	23,608 (92.7)
University/college degree, *n* (%)	103,270 (38.1)	42,608 (48.8)	1,920 (18.0)	56,092 (38.0)	2,650 (10.4)
No listed qualifications, *n* (%)	26,829 (9.9)	4,235 (4.8)	1,913 (17.9)	13,823 (9.4)	6,858 (26.9)
Never smoked, *n* (%)	155,916 (57.5)	55,264 (63.2)	5,805 (54.4)	82,097 (55.7)	12,750 (50.0)
Former smoker, *n* (%)	86,262 (31.8)	24,189 (27.7)	2,840 (26.6)	50,432 (34.2)	8,801 (34.5)
Current smoker, *n* (%)	28,793 (10.6)	7,923 (9.1)	2,021 (18.9)	14,922 (10.1)	3,927 (15.4)
Alcohol: daily/almost daily, *n* (%)	53,346 (19.7)	17,213 (19.7)	1,786 (16.7)	30,148 (20.4)	4,199 (16.5)
Alcohol: never, *n* (%)	16,436 (6.1)	4,639 (5.3)	797 (7.5)	8,957 (6.1)	2,043 (8.0)
Assessment center: England, *n* (%)	239,434 (88.4)	76,739 (87.8)	9,562 (89.6)	130,272 (88.3)	22,861 (89.7)
Assessment center: Scotland, *n* (%)	20,032 (7.4)	7,447 (8.5)	731 (6.9)	10,377 (7.0)	1,477 (5.8)
Assessment center: Wales, *n* (%)	11,505 (4.2)	3,190 (3.7)	373 (3.5)	6,802 (4.6)	1,140 (4.5)

Values are mean (standard deviation) or *n* (%). Columns refer to the complete-case adjusted population. Supplementary Tables S2A,B provide full distributions; Supplementary Tables S10, S13 describe participants with missing adjustment data. NR denotes values unavailable in the released aggregate tables, not zero. Higher frequency means usually/always undertaking heavy physical work; lower means never/rarely or sometimes. WHtR, waist-to-height ratio.

The full eligible population accrued 2,153 M51 events over 1,906,404 person-years (1.13 per 1,000 person-years); the nested subtype accrued 990 events over its separately defined 1,910,103 person-years (0.52 per 1,000 person-years). Median observed follow-up was 6.93 years. Primary complete-case models included 2,072 M51 and 951 subtype events. Outcome-specific totals are in Supplementary Table S1; Supplementary Table S11 gives event counts and person-time for the four exposure groups, distinguishing eligible and complete-case populations.

### 3.2 Continuous WHtR and workload frequency

Each 0.05 higher WHtR was associated with an M51 HR of 1.13 (95% CI 1.10–1.16); higher versus lower work frequency in the same continuous-WHtR model had HR 1.47 (1.31–1.65). The corresponding nested-subtype estimates were 1.14 (1.09–1.19) and 1.41 (1.19–1.66). The continuous-WHtR and original work-frequency estimates are presented in Table 2. Restricted cubic splines showed no clear evidence of nonlinearity (*p* = 0.263 and 0.308; Supplementary Figures S3, S4). Relative to never/rarely, the M51 HRs for sometimes, usually and always were 1.16, 1.48 and 1.62 after continuous-WHtR adjustment. Subtype point estimates were not strictly monotonic (Table 2; Supplementary Table S7; Supplementary Figure S5). A 0.05 WHtR increment and the four-group joint contrast are different comparisons; their HR magnitudes should not be equated.

**Table 2:** Joint exposure categories and incident hospital-recorded disease.

Outcome	Workload	WHtR	Model 1 HR (95% CI)	Model 2 HR (95% CI)
Intervertebral disc disorders	Lower	<0.5	1.00 (reference)	1.00 (reference)
	Higher	<0.5	1.74 (1.41–2.16)	1.47 (1.18–1.83)
	Lower	≥0.5	1.49 (1.34–1.66)	1.40 (1.25–1.56)
	Higher	≥0.5	2.50 (2.16–2.89)	2.04 (1.75–2.38)
Disc-related radiculopathy	Lower	<0.5	1.00 (reference)	1.00 (reference)
	Higher	<0.5	1.63 (1.18–2.25)	1.35 (0.97–1.88)
	Lower	≥0.5	1.50 (1.28–1.76)	1.41 (1.20–1.66)
	Higher	≥0.5	2.45 (1.98–3.04)	2.00 (1.59–2.50)

Both models use 270,971 complete cases, with 2,072 M51 and 951 radiculopathy-subtype events. Model 1 adjusts for age (natural spline) and sex, with regional strata. Model 2 additionally adjusts for ethnicity, education, deprivation, smoking and alcohol. Reference: lower heavy-work frequency/WHtR <0.5. The radiculopathy outcome is nested within M51 and is not an independent replication. Group events and person-time are given in Supplementary Table S11. CI, confidence interval; HR, hazard ratio.

### 3.3 Joint exposure categories and disease hazards

Compared with lower frequency/WHtR <0.5, adjusted M51 HRs were 1.47 (95% CI 1.18–1.83) for higher frequency/<0.5, 1.40 (1.25–1.56) for lower frequency/≥0.5 and 2.04 (1.75–2.38) for higher frequency/≥0.5 (Table 3; Figure 2). Corresponding subtype estimates were 1.35 (0.97–1.88), 1.41 (1.20–1.66) and 2.00 (1.59–2.50). The subtype comparison for higher frequency/<0.5 was imprecise and included the null; it describes a nested coding phenotype rather than replicating the M51 finding.

**Table 3:** Continuous WHtR and original work-frequency categories.

Exposure contrast	Disc disorders (M51)	Radiculopathy subtype
WHtR per 0.05 increase	1.13 (1.10–1.16)	1.14 (1.09–1.19)
Higher vs lower heavy-work frequency	1.47 (1.31–1.65)	1.41 (1.19–1.66)
Sometimes vs never/rarely	1.16 (1.04–1.29)	1.08 (0.92–1.27)
Usually vs never/rarely	1.48 (1.27–1.73)	1.50 (1.20–1.88)
Always vs never/rarely	1.62 (1.39–1.89)	1.39 (1.10–1.76)

*N* = 270,971; M51 events = 2,072; nested-subtype events = 951. WHtR and binary frequency estimates come from the same model. Four-level frequency estimates come from a separate model adjusting for continuous WHtR. All models include the Model 2 covariates and regional strata. Values are HRs (95% CIs); comparisons are not equivalent exposure increments.

**Figure 2:**
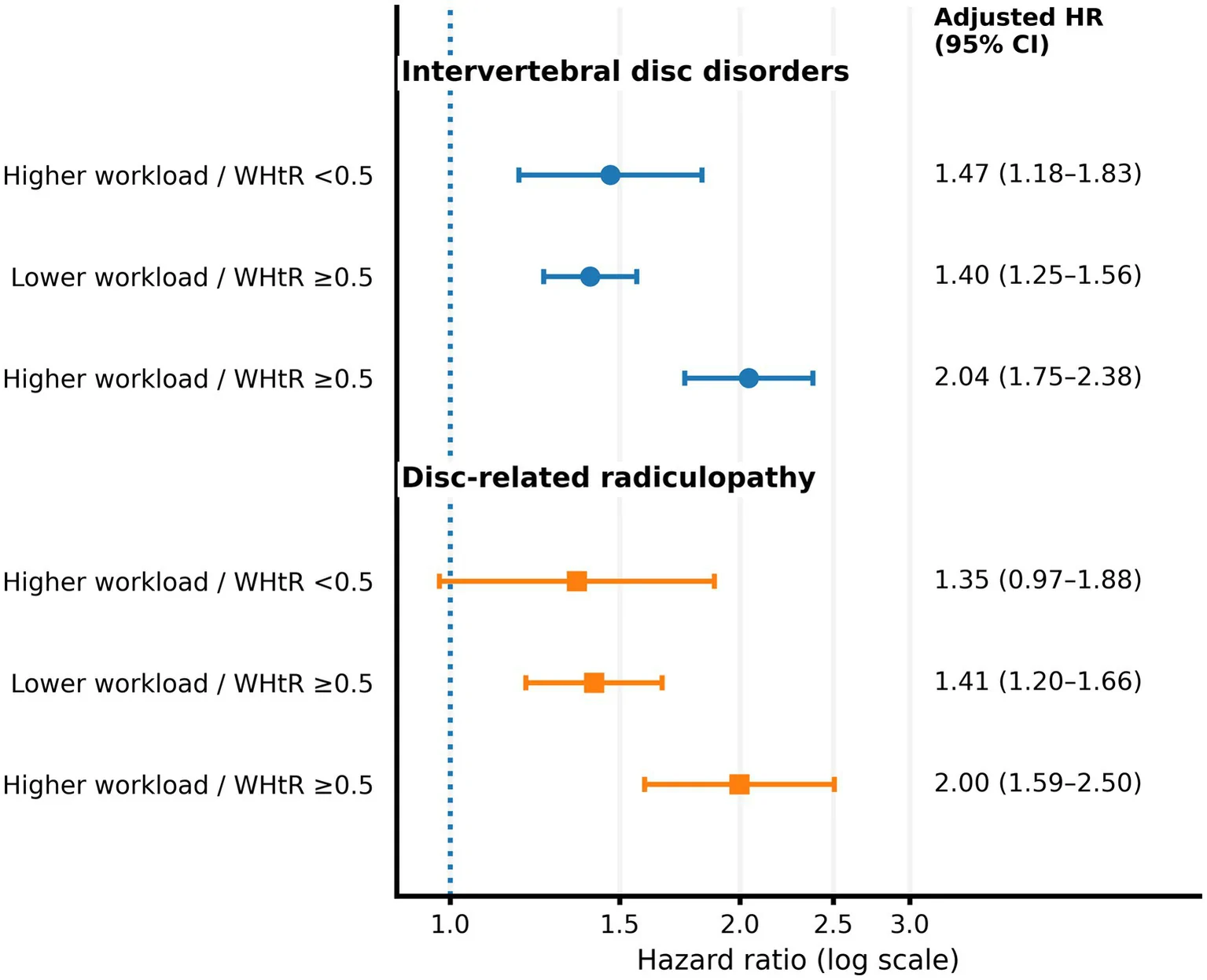
Joint occupational workload and waist-to-height ratio associations with hospital-recorded disease. Points and horizontal bars are adjusted hazard ratios and 95% confidence intervals. The reference is lower workload with WHtR <0.5. Both models use 270,971 complete cases, with 2,072 disc-disorder and 951 radiculopathy events. Adjustment includes age (natural spline), sex, ethnicity, education, deprivation, smoking, and alcohol intake, with regional baseline-hazard strata. The horizontal axis is logarithmic. WHtR, waist-to-height ratio; HR, hazard ratio; CI, confidence interval.

Exposure-factor proportional-hazards tests gave *p* = 0.407 for M51 and *p* = 0.264 for the subtype; global tests gave *p* = 0.211 and 0.738. Education in M51 (*p* = 0.045) and sex in the subtype (*p* = 0.043) showed nominal departures. Allowing their baseline hazards to vary by strata yielded higher-frequency/WHtR ≥0.5 HRs of 2.041 (1.751–2.379) and 1.994 (1.589–2.502), respectively, versus 2.042 and 1.996 in the primary models. The continuous WHtR estimates were also materially unchanged (Supplementary Table S12).

### 3.4 Standardized five-year risks and interaction

Standardized five-year M51 risks were 0.389% (95% CI 0.351–0.426) in the reference group and 0.791% (0.697–0.885) in the higher-frequency/WHtR ≥0.5 group (Table 4; Figure 3). The difference was 4.03 (3.01–5.04) per 1,000 people. Subtype risks were 0.174% (0.149–0.199) and 0.348% (0.285–0.410), a difference of 1.73 (1.07–2.40) per 1,000. These subtype risks are not added to the primary-outcome risks because the events overlap. Unadjusted estimates are reported separately in Supplementary Figures S1, S2 and Supplementary Table S5.

**Table 4:** Standardized five-year cumulative incidence and risk differences.

Outcome	Workload	WHtR	5-year risk, % (95% CI)	Risk difference per 1,000 (95% CI)
Intervertebral disc disorders	Lower	<0.5	0.389 (0.351–0.426)	Reference
	Higher	<0.5	0.569 (0.455–0.683)	1.81 (0.63–2.99)
	Lower	≥0.5	0.542 (0.507–0.577)	1.54 (1.05–2.02)
	Higher	≥0.5	0.791 (0.697–0.885)	4.03 (3.01–5.04)
Disc-related radiculopathy	Lower	<0.5	0.174 (0.149–0.199)	Reference
	Higher	<0.5	0.236 (0.164–0.308)	0.62 (-0.13–1.36)
	Lower	≥0.5	0.246 (0.222–0.269)	0.71 (0.39–1.04)
	Higher	≥0.5	0.348 (0.285–0.410)	1.73 (1.07–2.40)

All four exposure states are standardized over the same 270,971-person complete-case covariate distribution, using disease and competing-death cause-specific models. Adjustment matches Model 2. Confidence intervals incorporate the full influence-function covariance. Risk differences compare each group with lower workload/WHtR <0.5; they are not estimates of preventable disease under an intervention. CI, confidence interval; WHtR, waist-to-height ratio.

**Figure 3:**
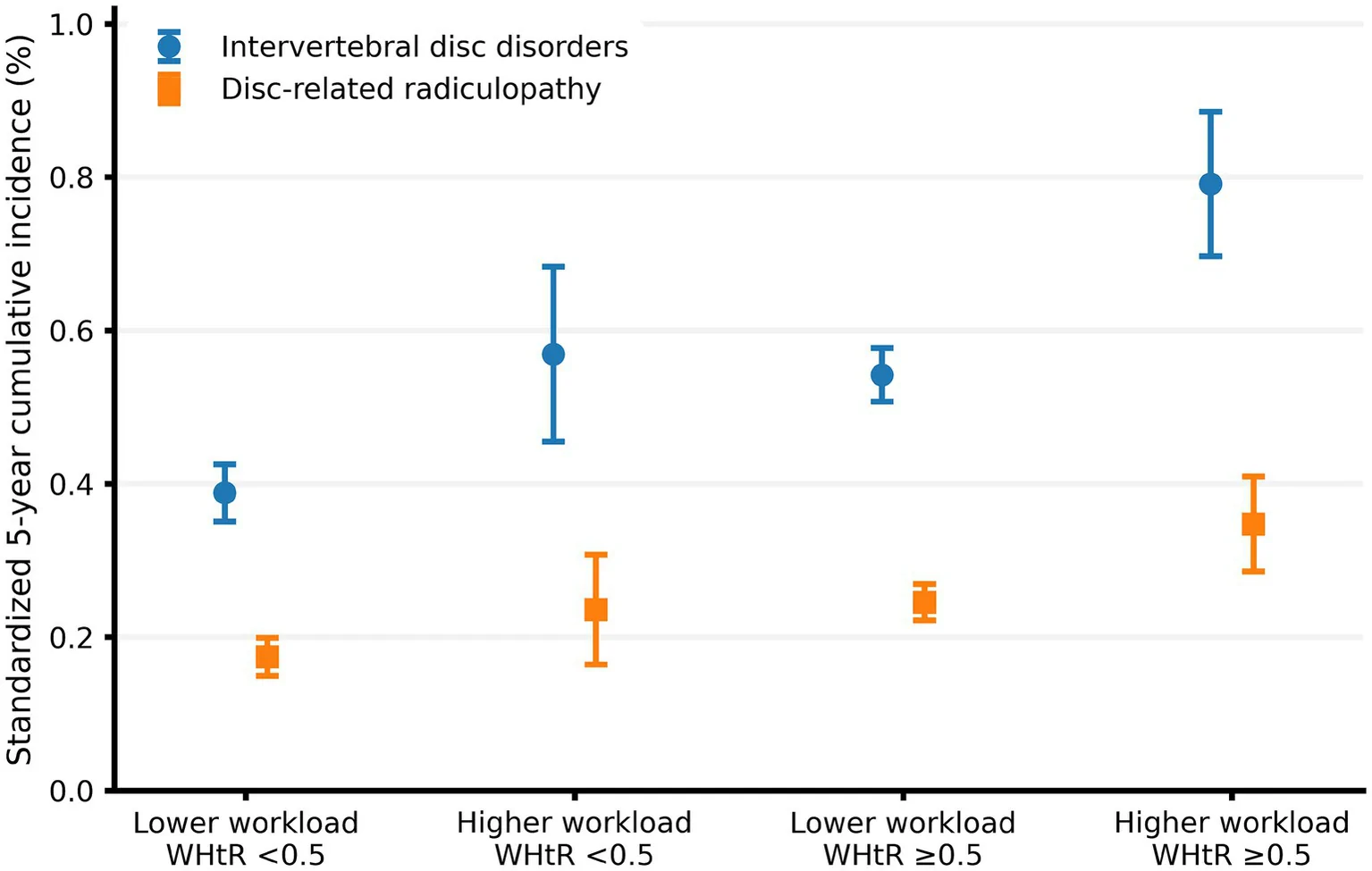
Standardized 5-year cumulative incidence by joint exposure category. Points and vertical bars show disease risks and 95% confidence intervals obtained from disease and competing-death cause-specific models. All four categories are standardized over the same 270,971-person complete-case covariate distribution. These are adjusted model-based risks, not crude event fractions or estimates of intervention efficacy. WHtR, waist-to-height ratio.

There was no clear evidence of multiplicative interaction (*p* = 0.975 and *p* = 0.815 for disc disorders and radiculopathy, respectively). The 5-year additive-interaction contrasts were 0.68 (95% CI − 0.80 to 2.16) and 0.40 (−0.54 to 1.35) additional events per 1,000 people, respectively (*p* = 0.368 and *p* = 0.405). These intervals include zero and do not support a claim of additional joint effects beyond the relevant scale-specific expectation (Supplementary Table S6).

### 3.5 Sensitivity and missing-data analyses

The joint-high group retained elevated point estimates in each specified sensitivity analysis (Figure 4; Supplementary Tables S3A, S3B). HRs for disc disorders and radiculopathy were 1.92 (1.60–2.30) and 1.92 (1.51–2.44) when only primary diagnoses were counted; 2.07 (1.74–2.46) and 2.10 (1.64–2.70) with two-year delayed entry; and 1.88 (1.43–2.46) and 1.82 (1.22–2.70) with five-year delayed entry. The median remaining observation time after five-year entry was approximately 1.94 years. In contrast, the higher-work-frequency/WHtR <0.5 comparison attenuated in the five-year delayed-entry risk sets: HR 1.00 (0.64–1.56) for disc disorders and 0.97 (0.51–1.85) for radiculopathy. These conditional populations differ from the baseline risk sets, so robustness of the joint-high contrast should not be generalized to every exposure contrast.

**Figure 4:**
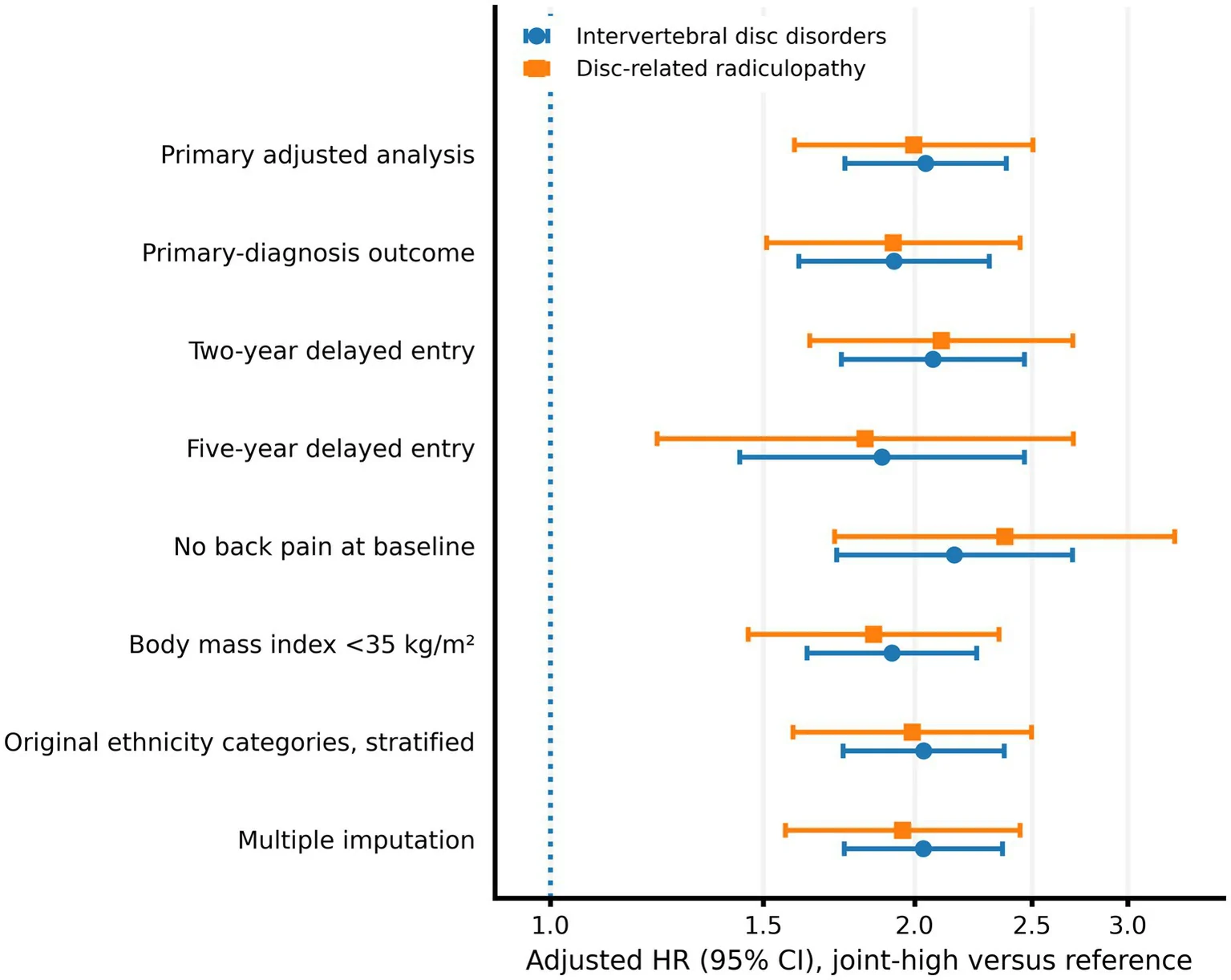
Specified sensitivity analyses for the higher-frequency/WHtR ≥0.5 versus reference association. Points and horizontal bars are hazard ratios and 95% confidence intervals relative to lower frequency/WHtR <0.5. Risk sets differ between analyses; denominators and all three group contrasts are given in Supplementary Tables S3A,B. Delayed entry begins two or five calendar years after baseline among participants alive, outcome-free and still followed. Imputation uses 20 completed datasets. The ethnicity sensitivity stratifies by the original six categories. Additional reviewer-requested models are reported in Supplementary Table S12. The horizontal axis is logarithmic. This display does not imply that every exposure contrast was stable.

The no-baseline-back-pain analysis gave higher-frequency/WHtR ≥0.5 HRs of 2.16 (1.72–2.70) for M51 and 2.37 (1.72–3.28) for the subtype. Restriction to England/Scotland assessment centers retained 259,466 complete cases, 1,995 M51 and 922 subtype events, with HRs of 2.035 (1.741–2.379) and 2.020 (1.605–2.541). In 267,227 complete cases with valid weekly work hours, adding hours changed the matched-sample HRs from 2.053 to 2.049 (1.756–2.391) for M51 and from 2.026 to 2.023 (1.612–2.540) for the subtype. Supplementary Table S12 reports all additional contrasts; continuous-WHtR sensitivities were performed for baseline-hazard stratification and regional restriction. The original six-category model gave HRs of 2.034 and 1.989 for this contrast, although its Chinese coefficient was unstable; the original-category stratified results were similar (Supplementary Table S15).

Missing adjustment data affected 5,946 participants (2.15%). Education had the highest variable-level missingness (1.43%). Crude event fractions were 1.36% versus 0.76% for M51, and 0.66% versus 0.35% for the subtype, in participants with incomplete versus complete adjustment data. These are descriptive fractions, not person-time-adjusted comparisons. The imputed higher-frequency/WHtR ≥0.5 HRs were 2.03 (1.75–2.36) and 1.95 (1.56–2.44). FMI for exposure coefficients ranged from 0.0011 to 0.0035; all imputed coefficients and covariate-specific FMI are provided in Supplementary Table S14 and its companion CSV. Observed and completed distributions appear in Supplementary Table S13, without treating their agreement as a test of missing at random.

## 4 Discussion

### 4.1 Principal findings

Baseline WHtR showed a graded positive association with subsequent hospital-recorded M51 disease: each 0.05 increase corresponded to an adjusted HR of 1.13, without clear evidence of nonlinearity. Higher versus lower heavy-work frequency in the same model had an HR of 1.47. The higher-frequency/WHtR ≥0.5 category had approximately twice the hazard of the reference category, corresponding to about four additional standardized events per 1,000 people over five years. Additional adjustment for weekly work hours, targeted baseline-hazard stratification and England/Scotland restriction changed these exposure estimates little. Together, the relative and absolute estimates describe occupational and anthropometric associations among adults employed at baseline.

The continuous and categorical analyses address complementary questions. Continuous WHtR describes a gradual association without assuming a disease threshold; the original four groups summarize joint exposure patterns. Multiplicative and additive interaction tests provided no clear evidence of an additional joint association on either scale. Persistence of the higher-frequency/≥0.5 contrast across sensitivity analyses did not extend uniformly to the higher-frequency/<0.5 contrast, which attenuated after five-year delayed entry.

### 4.2 Interpretation in relation to previous evidence

The association with heavy-work frequency is consistent with the broader evidence linking occupational loading to disc-related morbidity, although the exposure contrasts are not interchangeable (5–7). The EPILIFT study examined cumulative lumbar loading, whereas Euro and colleagues examined hospital-treated sciatica and joint body-mass-index–vibration categories (6, 7). In the latter study, task-specific lifting was positively associated with hospitalization, but the broad heavy/very-heavy-work category was inversely associated (6). This contrast suggests that a general work label combines task content, reporting and workforce selection differently from a task-specific lifting question. Wahlström and colleagues used a job-exposure matrix in 262,850 male construction workers followed for 33 years and studied surgically treated lumbar disc herniation (28). Our cohort adds women and men across occupations, measured WHtR and standardized absolute risks. It complements these studies rather than replacing their more detailed exposure assessment.

In our analysis, additional adjustment for weekly hours changed the joint-category estimate little, indicating that total working time did not explain that comparison in the matched subset. Hours do not, however, distinguish tasks performed within the working day. The single heavy-work item leaves posture, repetition and task arrangement unresolved. The next measurement step is therefore task characterization, not simply a more elaborate label for high workload.

Outcome definitions also matter when comparing studies. Imaging-defined degeneration is common even without symptoms (29, 30). Our M51 endpoint records diagnoses during inpatient or day-case care, including secondary diagnostic positions, and is shaped by symptoms, referral, access and coding. The resulting risk estimates concern this defined hospital endpoint, rather than all symptomatic disc disease in the community. The M51.1 analysis narrows the clinical coding phenotype; its shared events do not provide independent replication or a measure of symptom severity.

The anthropometric findings should likewise be interpreted against the exposure actually measured. Previous obesity studies used body mass index and outcomes ranging from sciatica to MRI-defined degeneration (9, 10). Here, continuous WHtR showed a graded association, while the higher-frequency/WHtR ≥0.5 category combined two exposure differences. A larger HR for that category is compatible with contributions from both exposures without an additional interaction on the tested scales. Mechanical loading and adiposity-related processes remain plausible explanations, informed by disc biology and the mixed genetic and environmental contributions described in twin studies (31, 32). Neither these mechanisms nor a WHtR effect independent of overall adiposity was tested in this cohort.

Occupational activity should also be distinguished from leisure-time activity. Their differing associations with cardiovascular events and mortality in the Copenhagen General Population Study emphasize the importance of activity domain and recovery (33). The present study concerns reported heavy-work frequency, not total physical activity. Separately measuring leisure exercise, recovery opportunities and specific occupational tasks would help determine whether workers with similar reported frequencies experience different combinations of physical demands.

### 4.3 Implications for occupational health

Joint consideration of occupational and anthropometric exposures makes the magnitude of hospital-recorded disc morbidity more explicit. Standardized five-year M51 risk was approximately 3.9 per 1,000 in the reference category and 7.9 per 1,000 in the higher-frequency/WHtR ≥0.5 category. Thus, a roughly twofold relative association coexisted with a modest absolute difference. These model-based exposure contrasts are not observed screening positive predictive values. Individual screening, fitness-for-work decisions and intervention benefits require additional evidence on prediction, decision thresholds and the effects of specific actions; they cannot be inferred from these associations alone.

A practical next step is a longitudinal study that repeats measurements of lifting load and frequency, bending, posture, vibration, work hours and recovery, alongside anthropometry, job changes and retirement. A suitable job-exposure matrix would complement task-level reports.

Intervention studies can then examine successive outcomes rather than assuming that an ergonomic change prevents hospital disease. Future trials should measure uptake, actual task modification, symptoms and work function, with sufficiently long follow-up for defined clinical events. This would connect workplace prevention to the hospital outcome quantified here.

### 4.4 Strengths and limitations

Strengths include the large baseline-employed cohort, measured anthropometry, explicit baseline exclusions, separate timing for the nested subtype and competing-risk absolute estimates. Continuous WHtR and the original work-frequency categories complement the four-group display. The original six-category ethnicity model, its instability and subsequent modification are reported together with the stratified alternative. All additional sensitivity contrasts are provided in Supplementary Table S12, including imprecise estimates.

Workload was a single baseline report, not a validated measure of biomechanical dose. Retirement, health-related task reduction, job change or continued work cannot be inferred for the full cohort. Repeat assessments within the historical window covered only a selected subset: approximately 11,200 participants attended the first repeat assessment and approximately 4,500 attended the first imaging assessment, with fewer valid work-frequency responses (Supplementary Table S16). Visits could occur after disease events and employment responses could combine paid work with retirement. These data were used descriptively, not to assign retirement dates or fit a time-updated causal model.

Healthy-worker selection operates both at enrollment and through subsequent retention in physically demanding jobs (34, 35). Symptomatic workers can change tasks or leave work, while healthier workers can remain exposed; entry into this study also required baseline employment. Reverse causation, selection and exposure misclassification therefore need not have a single net direction. The higher-frequency/<0.5 estimate attenuated to the null after five-year delayed entry, which is compatible with several explanations, including early symptomatic disease and selective retention, but does not distinguish them. Although the higher-frequency/≥0.5 contrast persisted, that does not eliminate these biases. Both delayed-entry analyses used common group rules; their later, selected risk sets and shorter remaining follow-up differ from the baseline population. Leisure-time activity, detailed job demands and other unmeasured factors remain possible confounders.

UK Biobank volunteers are not representative of all workers, and the predominantly White sample limits transportability (15). Missingness was associated descriptively with higher crude event fractions; concordance between complete-case and imputed estimates does not establish missing at random. The historical 2015 cutoff limits contemporary applicability and reduces follow-up relative to later releases. Same-cutoff England/Scotland results address dependence on assessment-center geography, but do not test a newer follow-up period or prove absence of underascertainment. Complete common coverage for a later period was not established for this extract, so follow-up was not extended. Remaining uncertainty also includes regional misclassification, incomplete loss information, coding error and model assumptions beyond sampling precision. No external validation cohort was analyzed.

## 5 Conclusion

Higher baseline WHtR and more frequent heavy physical work were associated with subsequent hospital-recorded intervertebral disc disorders. Continuous WHtR analysis supported a graded association, while the combined higher-frequency/WHtR ≥0.5 category showed an approximately twofold relative hazard and about four additional standardized events per 1,000 people over five years. There was no clear evidence of multiplicative or additive interaction. These findings quantify the associations and identify the need for repeated, task-specific exposure measurement before preventive interventions can be evaluated.

## Data Availability

Participant-level data are held by UK Biobank and are not redistributed by the authors. Researchers can apply for access through UK Biobank (https://www.ukbiobank.ac.uk/use-our-data/apply-for-access/). Operational definitions and aggregate study results accompany the article. Supplementary Code 1 supplies the executed model-fitting and imputation-inspection code with input specifications and software versions, and additional study-specific statistical code is available from the corresponding authors upon reasonable request.
