# Supplementary material for: Joint associations of baseline heavy physical work and waist-to-height ratio with hospital-recorded intervertebral disc disorders: a UK Biobank cohort study
Source: Front Public Health. 2026 Oct 28;14:2002097. doi: 10.3389/fpubh.2026.2002097 (PMC13752095; doi:10.3389/fpubh.2026.2002097)
Supplement: Supplementary file 1 [file Data_Sheet_1.docx]

Supplementary Material

Contents: Supplementary Methods A–G, operational Tables M1–M8; Tables S1–S16 (lettered panels where indicated); Figures S1–S5. Companion CSVs and Supplementary Code 1 provide selected detailed results and reviewer-requested analysis code. NR indicates cells not present as numerical values in the released aggregate data and is not zero.

# Supplementary Methods A. Records, timing and historical coverage

UK Biobank supplied linked participant records. The investigators joined these exports by the supplied identifier and paired diagnosis/date arrays by index. Hospital diagnoses came from admitted-patient data, including inpatient and day-case records; ordinary outpatient consultations were not systematically included. Summary diagnoses include primary and secondary diagnostic positions. No new person-to-registry linkage or participant-level clinical-chart validation was undertaken.

## Table M1. Sources and operational timing rules

| **Component** | **Actual rule** |
| --- | --- |
| Baseline assessment | Field 53, instance 0, in the 40129 baseline export; this assessment date is the time origin. Baseline age is 21022 and sex is 31. Follow-up measurements were not substituted for baseline exposures. |
| Hospital events | The selected 46925 summary pairs all-diagnosis ICD-10 field 41270 with first-date field 41280. Primary-diagnosis sensitivity uses 41202 with 41262. Matching uses the same array index within each pair; codes are not independently sorted and re-paired. |
| Historical disease evidence | Hospital M50/M51, ICD-9 field 41271/date 41281, and relevant OPCS-4 field 41272/date 41282 are checked for dates at or before baseline. Derived first-occurrence dates 131922 (M50) and 131924 (M51) add baseline-history evidence only; their mixed-source dates are not the incident hospital event dates. |
| Mortality source | Death/death_0413.txt: columns eid, ins_index, dsource, source and date_of_death. One uniform death-table version supplies follow-up dates. Source labels identify the provider and record format; neither the filename nor the maximum date is a release/completeness date. |
| Death comparisons | Repeated records are consolidated only when they have one distinct valid death date. Discordant dates within the selected table, conflicting old field-40000 dates, cross-version conflicts and old-snapshot-only deaths are not resolved by choosing the earliest value. Unresolved histories are excluded, not filled across sources. |
| Loss to follow-up | Baseline-export field 191 supplies known loss dates and 190 the reason. A known reason without a usable date is unresolved, except a death reason (1) resolved by the selected death table. Reason 5 denotes withdrawal from further linkage, not necessarily withdrawal of all research consent. Historical loss data may be incomplete. |
| Administrative end and ties | December 31, 2015 is the common administrative end. Stop at the earliest corresponding event, death, valid loss or administrative end. A disease record on the same day as death, loss or administrative end is counted as disease. Death before disease is the competing event. |
| Independent radiculopathy timing | For each participant, obtain the first qualifying M51.1-branch date independently. An earlier other M51 code does not censor radiculopathy follow-up. Entry precedes the event; nonpositive follow-up and unresolved qualifying dates do not become event-free time. |
| Delayed entry and region | Two/five calendar-year anniversaries define conditional entry among those alive, outcome-free and followed; start and stop remain measured from baseline. Field 54 assessment-center country supplies England/Scotland/Wales strata as a proxy, not continuous residential history. |

The recorded selection check used membership in the selected hospital export, including participants with no hospital diagnoses, rather than restricting to people who had ever been hospitalized. Individual last hospital dates were not censoring dates. The 8,695-person exclusion step combines several history/follow-up exclusions for disclosure protection and is not decomposed here.

## Table M2. Historical coverage evidence and its limits

| **Evidence** | **Interpretation** |
| --- | --- |
| Baseline export | Local provenance records identify 40129/ukb40129.html and export date January 17, 2020. This supplies baseline measurements and an older mortality comparator, not the death follow-up cutoff. |
| England: HES | The hospital-summary provenance identifies 46925/ukb46925.html, exported May 8, 2021. Recorded January/March 2021 summary-release chronology and December 2020 underlying updates support a window extending beyond 2015. These dates are export/release evidence, not a direct completeness certificate. |
| Scotland: SMR | The same dated summary and recorded release chronology support use of Scottish linked hospital records through the common pre-2016 window. Assessment-center country does not establish an individual’s provider throughout follow-up. |
| Wales: PEDW | The retained UK Biobank chronology records a July 2023 repair of missing Welsh diagnoses from 2016 onward. The older extract was therefore not extended across that later period. This fact alone does not establish complete pre-2016 ascertainment. |
| Corroborating checks | Existing all-cause provider-by-month checks show no empty month in 2006–2015 and high pre-2016 target-date concordance with a later raw hospital table. These are corroborating checks only; the later raw source was not merged into incident events. |
| Death linkage | The recorded historical UK Biobank death-linkage releases for England/Wales and Scotland, the selected table schema and comparisons with field 40000 support the bounded 2015 window. The exact extraction date and latest provider-specific completeness of death_0413.txt were not recovered. |
| Uncertainty retained | The provenance package contains source summaries rather than a formal provider-by-provider completeness certification. File-copy times and latest observed records were not treated as proof of coverage. |

Historical source: UK Biobank, Past data releases, https://community.ukbiobank.ac.uk/hc/en-gb/articles/26655145866269-Past-data-releases. The project used a fixed extract and a common pre-2016 observation window. A subsequent notification concerned missing Welsh diagnoses from 2016 onward; it does not show that all Welsh activity was absent. A same-cutoff England/Scotland assessment-center sensitivity is reported in Table S12. No new later cutoff was analyzed, because a common extended hospital/death/loss observation window was not established for this extract.

# Supplementary Methods B. Disease, surgery and baseline exclusions

## Table M3. Exact diagnostic and self-report code definitions

| **Role** | **Codes/rules actually used** |
| --- | --- |
| M50 baseline-history branch | M50, M500, M501, M502, M503, M508, M509 |
| M51 primary outcome and history branch | M51, M510, M511, M5116, M5119, M512, M513, M5137, M514, M518, M519 |
| M51.1 secondary outcome branch | M511, M5116, M5119 |
| ICD-9 history branch | 722, 7220, 7221, 7222, 7223, 7224, 7225, 7226, 7227, 7228, 7229; matched in 41271/41281. These were historical exclusion evidence, not incident ICD-10 outcomes. |
| Self-reported disease, field 20002 | All baseline arrays: 1312 (prolapsed/slipped disc), 1532 (disc problem), or 1533 (disc degeneration) supplied baseline disc-history exclusion evidence. Codes 1294 (back problem) and 1534 (back pain) were recorded as nonspecific symptoms, not automatically classified as disc disease. |
| Self-reported surgery, field 20004 | All baseline arrays: 1531 (discectomy) supplied disc-specific exclusion evidence. General spine/back surgery 1320 was not by itself classified as disc-specific surgery. |
| Derived first occurrence | 131922 and 131924 were used to identify at/before-baseline history or unresolved history dates. Corresponding source fields 131923/131925 were inspected for provenance, not used to replace incident hospital dates. |

ICD strings are shown without decimal points. Subordinate codes means membership in the UK Biobank coding hierarchy, not clinical validation. Only the listed branches were used; no additional ICD-10-CM extensions were introduced. General back pain, M47, M48, M54 or unlinked G55.1 were not incident outcomes. Self-report, procedure, date and censoring rules are separate parts of the cohort definition.

## Table M4. Procedure codes used for historical exclusion

| **Branch** | **Exact codes** | **Description** |
| --- | --- | --- |
| V29 | V29, V291, V292, V293, V294, V295, V298, V299 | V29 Primary excision of cervical intervertebral disc |
| V30 | V30, V301, V302, V303, V304, V305, V308, V309 | V30 Revisional excision of cervical intervertebral disc |
| V31 | V31, V311, V312, V314, V318, V319 | V31 Primary excision of thoracic intervertebral disc |
| V32 | V32, V321, V322, V324, V328, V329 | V32 Revisional excision of thoracic intervertebral disc |
| V33 | V33, V331, V332, V333, V334, V335, V336, V338, V339 | V33 Primary excision of lumbar intervertebral disc |
| V34 | V34, V341, V342, V343, V344, V345, V346, V348, V349 | V34 Revisional excision of lumbar intervertebral disc |
| V35 | V35, V351, V352, V358, V359 | V35 Excision of unspecified intervertebral disc |
| V36 | V36, V361, V362, V363, V368, V369 | V36 Prosthetic replacement of intervertebral disc |
| V51 | V51, V511, V518, V519 | V51 Other primary excision of lumbar intervertebral disc |
| V52 | V521, V522 | V52.1 Enzyme destruction of intervertebral disc; V52.2 Destruction of intervertebral disc NEC |
| V58 | V58, V581, V582, V583, V588, V589 | V58 Primary automated percutaneous mechanical excision of intervertebral disc |
| V59 | V59, V591, V592, V593, V598, V599 | V59 Revisional automated percutaneous mechanical excision of intervertebral disc |

The list is recovered from the project’s existing procedure dictionary extract and the original matching code. Codes in fields 41272/41282 were historical exclusion evidence when recorded at/before baseline. A recorded qualifying code with an unresolved date was not treated as absence of history. The procedure date is an episode/admission-derived summary date and was not represented as an exact operating-room timestamp. No incident surgery endpoint is added. Complete original dictionary labels are supplied in Definitions/Procedure_history_codes.csv.

# Supplementary Methods C. Employment, exposure and covariate routing

| **Variable** | **Operational definition** |
| --- | --- |
| Employment 6142/20119 | Paid employment/self-employment is code 1. All original 6142 arrays are considered. When 20119 is blank, use the original response; when a valid correction is present it takes precedence. A nonblank unknown/unwilling/invalid correction does not become definite employment by fallback. Simultaneous paid work and retirement is resolved by the recorded paid-work and correction rules. |
| Heavy physical work 816 | 1 never/rarely, 2 sometimes, 3 usually, 4 always. Higher frequency: 3/4; lower: 1/2. Codes −1/−3 and uninterpretable responses are missing, never lower exposure. This is current-main-job frequency, not measured lifting weight, mechanical dose or lifetime history. |
| Anthropometry 48/50; 21001 | Waist (48) and standing height (50) are in centimeters and from baseline; WHtR = waist/height. Both finite and positive; no winsorization. WHtR ≥0.5 is the higher category, informed by general health screening, not a disc-disease diagnostic boundary. BMI (21001) is descriptive and used only for the reported <35 kg/m² restriction. |
| Education 6138 | All baseline arrays; valid priorities are 1 degree > 2 A/AS > 5 or 6 vocational/professional > 3 GCSE/O > 4 CSE > −7 none listed. “None” with any positive qualification is contradictory. Code −3 (prefer not to answer), uninterpretable or contradictory responses are missing. The resulting six categories are nominal, not an official linear education score. |
| Ethnicity 21000 | Original parent classes 1 White, 2 Mixed, 3 Asian/Asian British, 4 Black/Black British, 5 Chinese, 6 Other (including applicable children). Following inspection of preliminary sparse-category estimates, 3 and 5 were pooled for adjustment only; no participants were removed. Original six-category ethnicity-by-region stratification is a sensitivity analysis. |
| Smoking/alcohol/deprivation | Smoking 20116: 0 never, 1 former, 2 current. Alcohol 1558: 1 daily/almost daily; 2 three/four times weekly; 3 once/twice weekly; 4 one–three times monthly; 5 special occasions; 6 never. Unknown/unwilling values are missing. Finite negative Townsend scores (189) are valid. |
| No-baseline-back-pain restriction | 6159 must have a valid baseline location response from 1–8 or −7, with no 4 (back pain) or 8 (pain throughout the body). −7 cannot coexist with a positive location. Unknown/unwilling or otherwise invalid responses fail the gate. Any 3571 response when 6159 does not select back pain is routing-inconsistent; blank 3571 is appropriate only with the valid negative location gate. A duration response of 0 does not negate current back pain. |

The original dated local plan specified the four groups and WHtR 0.5 before initial extraction (September 17, 2026, 00:09 +08:00). It was an internal record, not public preregistration. The ethnicity amendment was made after preliminary HRs, confidence intervals, P values and diagnostics had been reviewed. The Chinese category was numerically unstable, prompting pooling with Asian/Asian British and an original-six-category stratified sensitivity. These choices were not blinded to the preliminary results. The original coefficient model, pooled model and stratified model are displayed in Table S15. Later peer-review analyses were separate additions and did not replace the original estimates.

## Table M8. Covariate selection rationale

| **Covariate** | **Purpose in adjustment** |
| --- | --- |
| Age, sex | Demographic differences in work, anthropometry and recorded morbidity. |
| Ethnicity | Social/demographic context; analytic categories do not imply biological homogeneity. |
| Education, Townsend score | Socioeconomic patterning of work allocation, body size, healthcare access and disease recording. |
| Smoking, alcohol | Behavioral differences associated with employment and health. |
| Assessment-center country | Region-specific baseline hazards; not lifetime residence or provider geography. |
| Weekly hours (additional) | Sensitivity for one occupational characteristic, not a measure of total mechanical dose. |
| Leisure activity / detailed tasks | Not included in this analytic adjustment set; no claim that these variables do not exist in UK Biobank. |

# Supplementary Methods D. Exact spline and model specifications

| **Component** | **Retained implementation** |
| --- | --- |
| Age basis | Three centered natural-cubic-spline columns. Original specification: cr(age, df=3, constraints="center") − 1. Boundary knots: 38 and 71 years; interior knots: 49 and 60 years. The retained centering-constraint vector is (0.0847115573370195, 0.5095522575155498, 0.3867436039952593, 0.018992581152606127). The same existing columns were used across comparisons, rather than choosing new software defaults. |
| WHtR spline | Four knots: 0.4201183431952662, 0.4969325153374233, 0.550561797752809, 0.6582278481012658 (P5/P35/P65/P95). Hmisc::rcspline.eval with inclx=TRUE and norm=2 supplies three columns; overall and nonlinear tests use the appropriate joint covariance contrasts. |
| WHtR plotting and reference | Reference 0.5. Released grid spans P1=0.390874518427884 to P99=0.7329545454545454, using 101 evenly spaced points plus 0.5. |
| Hazard models | M1: exposure group + three age-basis columns + sex, with region-stratified baseline hazards. M2 adds five-category ethnicity, education, Townsend, smoking and alcohol. Tied times use Efron handling. Continuous WHtR models additionally adjust for binary heavy-work frequency; four-level frequency models instead adjust for WHtR per 0.05. |
| Reference categories | Joint exposure: lower frequency/WHtR <0.5. Region is a stratum. Covariate references are White ethnicity, degree education, never smoking and daily/almost-daily alcohol. Sex is the baseline binary field. The six-category ethnicity sensitivity stratifies on region × original ethnicity instead of estimating ethnicity coefficients. |

# Supplementary Methods E. Five-year cumulative incidence and uncertainty

Both disease and competing-death cause-specific models use the M2 covariates and regional strata in the same 270,971 complete cases. For each disease endpoint, competing death is death before that endpoint. riskRegression::CSC (fitter="coxph", ties="efron") combines both models, with disease as cause 1. Four exposure categories are evaluated at 1,826.25 days, retaining the same empirical distribution of all other covariates in every category.

The recorded riskRegression::ate call uses estimator="GFORMULA", treatment="group", cause=1, se=TRUE, iid=TRUE, known.nuisance=FALSE, and B=0; it does not use bootstrap resampling. Model-fitting uncertainty and the empirical target-distribution contribution are included in the influence functions. The four-by-four risk covariance is formed from the cross-products of the four corresponding influence-function columns and checked against the native standard errors. Risk-difference contrasts use this full covariance, as does F11−F10−F01+F00; normal-approximation contrast intervals use 1.96 standard errors. The recorded call seed is 2026091704.

Unadjusted Aalen–Johansen estimates use the eligible population with disease and competing death states and log-log intervals. Figures S1–S2 connect the released quarter-year grid from 0 to 5 years; they are not reconstructed event-time staircases. The at-risk counts in Table S5 are released counts. Standardization is associational and does not identify a specific intervention effect.

# Supplementary Methods F. Missing data, computation and available diagnostics

## Table M5. Actual chained-equation settings

| **Item** | **Recorded specification / limitation** |
| --- | --- |
| Imputed variables/methods | ethnicity5, education, smoking, alcohol: mice polyreg; Townsend: mice pmm. Other columns have empty methods and are not imputed. Missing counts: 833, 3,961, 744, 171 and 385, respectively; the union is 5,946 participants. |
| Datasets, iterations and seeds | 20 separate one-imputation chains, each with 20 iterations. Seeds are the consecutive integers 2026091704–2026091723, one per chain. The final execution record lists 12 workers. Earlier unscaled attempts were not used. |
| Starting predictor matrix | Constructed by mice::make.predictorMatrix on the 19-column input; diagonal entries zero and rows for all non-imputed variables zero. Each of the five imputed rows initially uses every other column. Columns are listed in Table M6 and the full code-derived starting matrix is supplied as Definitions/MI_initial_predictor_matrix.csv. |
| Survival auxiliaries | For each of M51 and M511, include disease and competing-death indicators and the corresponding cumulative baseline hazards. Each auxiliary is from an intercept-only cause-specific Cox fit with Breslow ties, evaluated at that outcome-specific stop time using the right-continuous cumulative-hazard lookup. Thus four indicators and four cumulative-hazard auxiliaries augment the imputation model. |
| Auxiliary scaling | The four cumulative hazards were centered and scaled to unit standard deviation before the final chains, following a low-variance-filtering probe. Actual means/SDs are supplied in Definitions/MI_auxiliary_scaling.csv. This recorded amendment preceded completion of the final MI estimates; it did not alter the outcomes, exposures, risk times or chain seeds. |
| Explicit fitting options | nnet.MaxNWts=10000; nnet.maxit=100; remove.constant=TRUE; remove.collinear=TRUE; printFlag=FALSE. PMM donor count and matching parameters were not explicitly set in the recorded call. Their realized runtime values are not exported; no guessed defaults are reported here. |
| Final predictor specification | Final predictor matrices for all 20 saved chains retained the initial predictor specification, including all four scaled cumulative-hazard auxiliaries and both disease/competing-death indicators. Saved loggedEvents entries were empty. Final predictor inclusion is provided with Table S14; these computational records do not establish the missing-at-random assumption. |
| Pooling | Each completed dataset used the same M2 Cox model. Rubin pooling is on log-HR: T = mean(U) + (1+1/20) × between-imputation covariance. mice::pool.scalar uses Barnard–Rubin degrees of freedom, with complete-data degrees of freedom events−24: 2,129 for M51 and 966 for M511. Reported intervals and P values use the pooled t distribution, not averages of HRs or P values. |

## Table M6. Imputation columns and existing chain diagnostics

| **Column group** | **Columns** |
| --- | --- |
| Exposure/covariates | group, age_ns1, age_ns2, age_ns3, sex, region, ethnicity5, education, townsend, smoking, alcohol |
| M51 survival augmentation | M51_event, M51_event_NA, M51_death_event, M51_death_event_NA |
| M51.1 survival augmentation | M511_event, M511_event_NA, M511_death_event, M511_death_event_NA |

The final predictor matrices from all 20 saved imputation chains retained the exposure/covariate terms, the M51 and M51.1 event indicators, their respective competing-death indicators, and four scaled Nelson–Aalen-type cumulative-hazard auxiliaries. For each target, final inclusion matched the initial predictor specification; saved loggedEvents entries were empty. NA within auxiliary names denotes Nelson–Aalen augmentation, not a missing observation. The final inclusion table accompanies Table S14.

| **Imputed variable** | **Split diagnostic** | **Chains / splits** |
| --- | --- | --- |
| ethnicity5 | 0.9938 | 20 / 40 |
| education | 0.9634 | 20 / 40 |
| townsend | 0.9599 | 20 / 40 |
| smoking | 0.9908 | 20 / 40 |
| alcohol | 1.0087 | 20 / 40 |

Each of the 20 saved objects contained one completed dataset after 20 iterations. The reported diagnostic splits iterations 11–20 across the chains and compares between/within-chain variation in chain means. The saved observed covariate values were retained. These diagnostics characterize the stored computation, not the validity of the missing-at-random assumption. Rubin variance, Barnard–Rubin degrees of freedom and FMI are reported for all pooled coefficients in the companion Table S14 CSV. No imputed participant-level datasets are distributed.

## Table M7. Actual analysis software versions

| **Software** | **Recorded version** |
| --- | --- |
| R | 4.5.3 (2026-03-11 ucrt), Windows x64 |
| broom | 1.0.13 |
| data.table | 1.18.6.1 |
| Hmisc | 5.3-0 |
| jsonlite | 2.0.0 |
| mice | 3.19.0 |
| prodlim | 2026.03.11 |
| riskRegression | 2026.03.11 |
| rms | 8.1-1 |
| nnet | 7.3-20 |
| splines | 4.5.3 |
| survival | 3.8-6 |
| Python / statsmodels | Python 3.12; statsmodels 0.14.6 for independent computational comparisons |

Versions are listed in Table M7. ChatGPT assisted with text organization, drafting, editing and aggregate visualization; Codex assisted with statistical-code implementation, debugging and aggregate preparation. Historical model identifiers were not consistently recorded. Scientific decisions, interpretation and submission responsibility remain with the authors. The reviewer-requested model-fitting functions and imputation-inspection scripts are provided in Supplementary Code 1.

Table S8 reports the original proportional-hazards tests. Table S12 reports sensitivities allowing education-specific or sex-specific baseline hazards. Table S15 documents the ethnicity amendment and all three specifications.

## Disclosure and source availability

Aggregate results and operational definitions accompany the article. Supplementary Code 1 provides the sensitivity-model and imputation-inspection scripts, required aggregate reference coefficients and a prepared-data schema. Additional statistical code is available from the corresponding authors on reasonable request. Participant data are not redistributed. Historical numerical cells not released remain NR.

# Supplementary Methods G. Reviewer-requested sensitivity and descriptive analyses

All additional fits retained the original definitions, 2015 cutoff, age basis, exposure references and Efron handling of ties. For M51, the PH sensitivity stratified by region × education and omitted education fixed effects; for the subtype it stratified by region × sex and omitted sex fixed effects. Each was fitted for categorical and continuous exposures. Country restriction used England/Scotland assessment centers, not inferred lifetime residence. These specifications were recorded before inspecting their new estimates.

Work hours (field 767, baseline) describe typical weekly hours in the main job, excluding travel. Valid 0–160-hour responses were represented by a single linear hours/10 term. Both the unextended and hours-adjusted models used exactly 267,227 complete cases with valid hours. Exposure estimates from identical samples are presented side by side; their change is descriptive and is not an independent-samples test or proof of confounding control.

Repeated assessments used field 53 dates and corresponding work/employment responses within baseline < repeat ≤ minimum(death, loss, December 31, 2015). Disease-free status was not required for this descriptive inventory. People can appear at both visits and can select both paid work and retirement. These observations do not supply whole-cohort employment histories, exact retirement dates or sufficient time-varying confounder histories for inverse-probability models. Counts retained as intervals are shown without substituting interval midpoints.

# Supplementary Table S1. Follow-up and outcome accrual

| **Outcome** | **Population** | **N** | **Events** | **Competing deaths** | **Person-years** | **Observed follow-up, median (IQR), years** | **Events per 1,000 person-years** |
| --- | --- | --- | --- | --- | --- | --- | --- |
| Intervertebral disc disorders | Eligible | 276,917 | 2,153 | 4,233 | 1,906,404 | 6.93 (6.21–7.61) | 1.13 |
| Intervertebral disc disorders | Complete cases | 270,971 | 2,072 | 4,114 | 1,860,012 | 6.92 (6.21–7.59) | 1.11 |
| Disc-related radiculopathy | Eligible | 276,917 | 990 | 4,266 | 1,910,103 | 6.93 (6.22–7.61) | 0.52 |
| Disc-related radiculopathy | Complete cases | 270,971 | 951 | 4,146 | 1,863,576 | 6.92 (6.22–7.59) | 0.51 |

Follow-up ends at the first relevant event, death, valid loss-to-follow-up date, or December 31, 2015. These medians summarize observed individual durations, not reverse-Kaplan–Meier potential follow-up. Death counts represent deaths before the corresponding disease event. IQR, interquartile range.

# Supplementary Table S2A. Complete-case baseline distributions

| **Characteristic** | **Overall n = 270,971** | **Lower workload WHtR <0.5 n = 87,376** | **Higher workload WHtR <0.5 n = 10,666** | **Lower workload WHtR ≥0.5 n = 147,451** | **Higher workload WHtR ≥0.5 n = 25,478** |
| --- | --- | --- | --- | --- | --- |
| Age, years | 52.7 (7.1) | 51.3 (6.8) | 51.1 (7.0) | 53.6 (7.1) | 53.1 (7.1) |
| WHtR | 0.529 (0.073) | 0.456 (0.030) | 0.459 (0.029) | 0.569 (0.056) | 0.572 (0.056) |
| BMI, kg/m² | 27.2 (4.7) | NR | 23.4 (2.2) | NR | 29.4 (4.2) |
| Townsend index | -1.35 (3.00) | -1.59 (2.89) | -0.90 (3.15) | -1.38 (2.99) | -0.51 (3.22) |
| Women | 141,310 (52.1) | 63,063 (72.2) | 6,089 (57.1) | 64,423 (43.7) | 7,735 (30.4) |
| Men | 129,661 (47.9) | 24,313 (27.8) | 4,577 (42.9) | 83,028 (56.3) | 17,743 (69.6) |
| Workload: never/rarely | 176,878 (65.3) | 68,763 (78.7) | — | 108,115 (73.3) | — |
| Workload: sometimes | 57,949 (21.4) | 18,613 (21.3) | — | 39,336 (26.7) | — |
| Workload: usually | 18,305 (6.8) | — | 5,558 (52.1) | — | 12,747 (50.0) |
| Workload: always | 17,839 (6.6) | — | 5,108 (47.9) | — | 12,731 (50.0) |
| Ethnicity: White | 255,422 (94.3) | 83,562 (95.6) | 10,039 (94.1) | 138,213 (93.7) | 23,608 (92.7) |
| Ethnicity: Asian/Asian British or Chinese | 6,261 (2.3) | 1,499 (1.7) | 202 (1.9) | 3,783 (2.6) | 777 (3.0) |
| Ethnicity: Black/Black British | 4,946 (1.8) | 1,082 (1.2) | 201 (1.9) | 3,062 (2.1) | 601 (2.4) |
| Ethnicity: Mixed | 1,900 (0.7) | 664 (0.8) | 94 (0.9) | 976 (0.7) | 166 (0.7) |
| Ethnicity: Other | 2,442 (0.9) | 569 (0.7) | 130 (1.2) | 1,417 (1.0) | 326 (1.3) |

Continuous values are mean (SD); categorical values are n (%). These are complete-case denominators. BMI is descriptive and has 172 missing observations overall. NR means suppressed/not released and must not be interpreted as zero; — denotes a workload category incompatible with the group definition. WHtR, waist-to-height ratio.

# Supplementary Table S2B. Complete-case baseline distributions

| **Characteristic** | **Overall n = 270,971** | **Lower workload WHtR <0.5 n = 87,376** | **Higher workload WHtR <0.5 n = 10,666** | **Lower workload WHtR ≥0.5 n = 147,451** | **Higher workload WHtR ≥0.5 n = 25,478** |
| --- | --- | --- | --- | --- | --- |
| Education: degree | 103,270 (38.1) | 42,608 (48.8) | 1,920 (18.0) | 56,092 (38.0) | 2,650 (10.4) |
| Education: A/AS level | 33,366 (12.3) | 12,027 (13.8) | 1,087 (10.2) | 18,688 (12.7) | 1,564 (6.1) |
| Education: vocational/professional | 62,079 (22.9) | 14,969 (17.1) | 3,498 (32.8) | 34,534 (23.4) | 9,078 (35.6) |
| Education: GCSE/O level | 33,088 (12.2) | 10,499 (12.0) | 1,319 (12.4) | 18,225 (12.4) | 3,045 (12.0) |
| Education: CSE | 12,339 (4.6) | 3,038 (3.5) | 929 (8.7) | 6,089 (4.1) | 2,283 (9.0) |
| Education: none listed | 26,829 (9.9) | 4,235 (4.8) | 1,913 (17.9) | 13,823 (9.4) | 6,858 (26.9) |
| Smoking: never | 155,916 (57.5) | 55,264 (63.2) | 5,805 (54.4) | 82,097 (55.7) | 12,750 (50.0) |
| Smoking: former | 86,262 (31.8) | 24,189 (27.7) | 2,840 (26.6) | 50,432 (34.2) | 8,801 (34.5) |
| Smoking: current | 28,793 (10.6) | 7,923 (9.1) | 2,021 (18.9) | 14,922 (10.1) | 3,927 (15.4) |
| Alcohol: daily/almost daily | 53,346 (19.7) | 17,213 (19.7) | 1,786 (16.7) | 30,148 (20.4) | 4,199 (16.5) |
| Alcohol: 3–4 times/week | 66,986 (24.7) | 23,543 (26.9) | 2,215 (20.8) | 35,959 (24.4) | 5,269 (20.7) |
| Alcohol: once/twice/week | 74,989 (27.7) | 24,212 (27.7) | 3,144 (29.5) | 39,936 (27.1) | 7,697 (30.2) |
| Alcohol: 1–3 times/month | 32,062 (11.8) | 10,061 (11.5) | 1,397 (13.1) | 17,482 (11.9) | 3,122 (12.3) |
| Alcohol: special occasions only | 27,152 (10.0) | 7,708 (8.8) | 1,327 (12.4) | 14,969 (10.2) | 3,148 (12.4) |
| Alcohol: never | 16,436 (6.1) | 4,639 (5.3) | 797 (7.5) | 8,957 (6.1) | 2,043 (8.0) |
| Assessment center: England | 239,434 (88.4) | 76,739 (87.8) | 9,562 (89.6) | 130,272 (88.3) | 22,861 (89.7) |
| Assessment center: Scotland | 20,032 (7.4) | 7,447 (8.5) | 731 (6.9) | 10,377 (7.0) | 1,477 (5.8) |
| Assessment center: Wales | 11,505 (4.2) | 3,190 (3.7) | 373 (3.5) | 6,802 (4.6) | 1,140 (4.5) |

Continuous values are mean (SD); categorical values are n (%). These are complete-case denominators. BMI is descriptive and has 172 missing observations overall. NR means suppressed/not released and must not be interpreted as zero; — denotes a workload category incompatible with the group definition. WHtR, waist-to-height ratio.

# Supplementary Table S3A. Sensitivity analyses — intervertebral disc disorders

| **Analysis** | **N** | **Events** | **Higher workload / WHtR <0.5 HR (95% CI)** | **Lower workload / WHtR ≥0.5 HR (95% CI)** | **Higher workload / WHtR ≥0.5 HR (95% CI)** |
| --- | --- | --- | --- | --- | --- |
| Primary adjusted | 270,971 | 2,072 | 1.47 (1.18–1.83) | 1.40 (1.25–1.56) | 2.04 (1.75–2.38) |
| Primary diagnosis | 270,971 | 1,500 | 1.42 (1.10–1.84) | 1.39 (1.22–1.58) | 1.92 (1.60–2.30) |
| Two-year delayed entry | 269,707 | 1,599 | 1.39 (1.08–1.80) | 1.41 (1.24–1.60) | 2.07 (1.74–2.46) |
| Five-year delayed entry | 266,505 | 660 | 1.00 (0.64–1.56) | 1.37 (1.12–1.67) | 1.88 (1.43–2.46) |
| No baseline back pain | 205,497 | 986 | 1.48 (1.07–2.04) | 1.34 (1.14–1.56) | 2.16 (1.72–2.70) |
| BMI <35 kg/m² | 253,564 | 1,861 | 1.44 (1.16–1.80) | 1.34 (1.20–1.51) | 1.92 (1.63–2.25) |
| Original ethnicity, stratified | 270,971 | 2,072 | 1.47 (1.18–1.83) | 1.39 (1.25–1.56) | 2.03 (1.74–2.37) |

Reference: lower workload/WHtR <0.5. All models use the primary adjustment variables except the ethnicity-stratified model, which controls the original six categories through the baseline-hazard strata. Each row is a distinct analytic risk set. No significance-based filtering was applied. Five-year delayed entry leaves about 1.94 years of median subsequent observation.

# Supplementary Table S3B. Sensitivity analyses — disc-related radiculopathy

| **Analysis** | **N** | **Events** | **Higher workload / WHtR <0.5 HR (95% CI)** | **Lower workload / WHtR ≥0.5 HR (95% CI)** | **Higher workload / WHtR ≥0.5 HR (95% CI)** |
| --- | --- | --- | --- | --- | --- |
| Primary adjusted | 270,971 | 951 | 1.35 (0.97–1.88) | 1.41 (1.20–1.66) | 2.00 (1.59–2.50) |
| Primary diagnosis | 270,971 | 835 | 1.24 (0.87–1.78) | 1.37 (1.15–1.63) | 1.92 (1.51–2.44) |
| Two-year delayed entry | 269,992 | 766 | 1.26 (0.87–1.84) | 1.43 (1.19–1.72) | 2.10 (1.64–2.70) |
| Five-year delayed entry | 267,275 | 319 | 0.97 (0.51–1.85) | 1.45 (1.09–1.92) | 1.82 (1.22–2.70) |
| No baseline back pain | 205,497 | 465 | 1.31 (0.80–2.15) | 1.42 (1.13–1.79) | 2.37 (1.72–3.28) |
| BMI <35 kg/m² | 253,564 | 852 | 1.32 (0.95–1.84) | 1.34 (1.13–1.58) | 1.85 (1.46–2.35) |
| Original ethnicity, stratified | 270,971 | 951 | 1.36 (0.98–1.88) | 1.41 (1.19–1.66) | 1.99 (1.59–2.50) |

Reference: lower workload/WHtR <0.5. All models use the primary adjustment variables except the ethnicity-stratified model, which controls the original six categories through the baseline-hazard strata. Each row is a distinct analytic risk set. No significance-based filtering was applied. Five-year delayed entry leaves about 1.94 years of median subsequent observation.

# Supplementary Table S4. Complete-case and multiple-imputation estimates

| **Outcome** | **Exposure category** | **Complete-case HR (95% CI)** | **Multiple-imputation HR (95% CI)** |
| --- | --- | --- | --- |
| Intervertebral disc disorders | Higher workload / WHtR <0.5 | 1.47 (1.18–1.83) | 1.44 (1.16–1.78) |
| Intervertebral disc disorders | Lower workload / WHtR ≥0.5 | 1.40 (1.25–1.56) | 1.40 (1.25–1.56) |
| Intervertebral disc disorders | Higher workload / WHtR ≥0.5 | 2.04 (1.75–2.38) | 2.03 (1.75–2.36) |
| Disc-related radiculopathy | Higher workload / WHtR <0.5 | 1.35 (0.97–1.88) | 1.33 (0.96–1.84) |
| Disc-related radiculopathy | Lower workload / WHtR ≥0.5 | 1.41 (1.20–1.66) | 1.40 (1.20–1.65) |
| Disc-related radiculopathy | Higher workload / WHtR ≥0.5 | 2.00 (1.59–2.50) | 1.95 (1.56–2.44) |

Reference: lower work frequency/WHtR <0.5. Complete-case N = 270,971, with 2,072 M51 and 951 subtype events. Multiple-imputation N = 276,917, with 2,153 and 990 events. Twenty completed datasets were combined on the log-HR scale, imputing missing adjustment covariates only. FMI and final imputation-predictor inclusion are added in Table S14; observed/completed characteristics and missingness are in Table S13.

# Supplementary Table S5. Unadjusted five-year cumulative incidence

| **Outcome** | **Workload** | **WHtR** | **5-year risk, % (95% CI)** | **At risk at 5 years** |
| --- | --- | --- | --- | --- |
| Intervertebral disc disorders | Lower | <0.5 | 0.380 (0.341–0.422) | 87,689 |
|  | Higher | <0.5 | 0.714 (0.570–0.886) | 10,735 |
|  | Lower | ≥0.5 | 0.537 (0.502–0.575) | 148,125 |
|  | Higher | ≥0.5 | 0.873 (0.766–0.991) | 25,775 |
| Disc-related radiculopathy | Lower | <0.5 | 0.177 (0.151–0.206) | 87,866 |
|  | Higher | <0.5 | 0.311 (0.220–0.431) | 10,779 |
|  | Lower | ≥0.5 | 0.237 (0.213–0.263) | 148,571 |
|  | Higher | ≥0.5 | 0.395 (0.325–0.477) | 25,900 |

Aalen–Johansen estimates in the eligible population with death as a competing event. Confidence intervals use the log-log transformation. These estimates differ in both adjustment and target population from Table 3; they must not be substituted for standardized complete-case risks.

# Supplementary Table S6. Interaction estimates on two scales

| **Outcome** | **Scale** | **Estimate (95% CI)** | **P** |
| --- | --- | --- | --- |
| Intervertebral disc disorders | Multiplicative | 0.996 (0.777–1.276) | 0.975 |
|  | Additive, per 1,000 | 0.681 (-0.801–2.163) | 0.368 |
| Disc-related radiculopathy | Multiplicative | 1.045 (0.721–1.515) | 0.815 |
|  | Additive, per 1,000 | 0.402 (-0.545–1.348) | 0.405 |

Multiplicative estimate = exp(β11 − β10 − β01), with null value 1. Additive estimate = [F11(5) − F10(5) − F01(5) + F00(5)] × 1,000, with null value 0; F denotes standardized 5-year cumulative incidence. Complete covariance matrices were used. These are secondary comparisons. Non-significant tests are not proof of exactly zero interaction.

# Supplementary Table S7. Continuous anthropometry and original workload levels

| **Outcome** | **Contrast** | **Adjusted HR (95% CI)** | **P** |
| --- | --- | --- | --- |
| Intervertebral disc disorders | WHtR, per 0.05 increase | 1.13 (1.10–1.16) | <0.001 |
|  | Sometimes vs never/rarely | 1.16 (1.04–1.29) | 0.008 |
|  | Usually vs never/rarely | 1.48 (1.27–1.73) | <0.001 |
|  | Always vs never/rarely | 1.62 (1.39–1.89) | <0.001 |
| Disc-related radiculopathy | WHtR, per 0.05 increase | 1.14 (1.09–1.19) | <0.001 |
|  | Sometimes vs never/rarely | 1.08 (0.92–1.27) | 0.345 |
|  | Usually vs never/rarely | 1.50 (1.20–1.88) | <0.001 |
|  | Always vs never/rarely | 1.39 (1.10–1.76) | 0.005 |

Continuous WHtR models adjust for binary work frequency and primary covariates; four-level frequency models adjust for continuous WHtR. N = 270,971; M51/subtype events = 2,072/951. Spline nonlinearity P = 0.263/0.308. Main Table 2B also displays these estimates. Categories measure reported frequency, not equal increments in biomechanical dose.

# Supplementary Table S8. Main-model proportional-hazards tests

| **Outcome** | **Term** | **df** | **χ²** | **P** |
| --- | --- | --- | --- | --- |
| Intervertebral disc disorders | Joint exposure | 3 | 2.90 | 0.407 |
|  | Age (joint spline) | 3 | 1.04 | 0.791 |
|  | Sex | 1 | 2.71 | 0.100 |
|  | Ethnicity | 4 | 6.43 | 0.169 |
|  | Education | 5 | 11.35 | 0.045 |
|  | Deprivation | 1 | 0.06 | 0.803 |
|  | Smoking | 2 | 1.89 | 0.389 |
|  | Alcohol | 5 | 2.83 | 0.726 |
|  | Global | 24 | 29.24 | 0.211 |
| Disc-related radiculopathy | Joint exposure | 3 | 3.98 | 0.264 |
|  | Age (joint spline) | 3 | 1.65 | 0.648 |
|  | Sex | 1 | 4.10 | 0.043 |
|  | Ethnicity | 4 | 0.68 | 0.954 |
|  | Education | 5 | 1.70 | 0.889 |
|  | Deprivation | 1 | 0.00 | 0.994 |
|  | Smoking | 2 | 3.75 | 0.153 |
|  | Alcohol | 5 | 4.09 | 0.537 |
|  | Global | 24 | 19.26 | 0.738 |

Schoenfeld-residual tests with Kaplan–Meier transformation, reported at factor level and globally. Main models use 270,971 complete cases. Diagnostic P values do not measure confounding or prove that model specification is correct.

# Supplementary Table S9. Diagnosis composition at the first qualifying date

| **Outcome** | **Population** | **Code** | **Participants** |
| --- | --- | --- | --- |
| Intervertebral disc disorders | Eligible | M510 | 60 |
| Intervertebral disc disorders | Eligible | M511 | 873 |
| Intervertebral disc disorders | Eligible | M512 | 653 |
| Intervertebral disc disorders | Eligible | M513 | 627 |
| Intervertebral disc disorders | Eligible | M514 | 12 |
| Intervertebral disc disorders | Eligible | M518 | 31 |
| Intervertebral disc disorders | Eligible | M519 | 27 |
| Intervertebral disc disorders | Complete cases | M510 | NR |
| Intervertebral disc disorders | Complete cases | M511 | 837 |
| Intervertebral disc disorders | Complete cases | M512 | 635 |
| Intervertebral disc disorders | Complete cases | M513 | 601 |
| Intervertebral disc disorders | Complete cases | M514 | 12 |
| Intervertebral disc disorders | Complete cases | M518 | NR |
| Intervertebral disc disorders | Complete cases | M519 | NR |
| Disc-related radiculopathy | Eligible | M511 | 990 |
| Disc-related radiculopathy | Complete cases | M511 | 951 |

Codes can overlap on the same person’s first qualifying date and counts need not sum to unique events. NR denotes protected values and is not zero. The M51.1 branch was derived independently of the first overall M51 date. Hospital records identify coded disease, not independently verified imaging or neurological findings.

# Supplementary Table S10. Complete-case and non-complete-case participants

| **Characteristic** | **Complete cases n = 270,971** | **Missing adjustment data n = 5,946** |
| --- | --- | --- |
| Age, years | 52.7 (7.1) | 53.0 (7.3) |
| WHtR | 0.529 (0.073) | 0.541 (0.074) |
| BMI, kg/m² | 27.2 (4.7) | 27.6 (4.8) |
| Townsend index | -1.35 (3.00) | -0.58 (3.27) |
| Women | 141,310 (52.1) | 2,948 (49.6) |
| Men | 129,661 (47.9) | 2,998 (50.4) |
| Workload: never/rarely | 176,878 (65.3) | 3,207 (53.9) |
| Workload: sometimes | 57,949 (21.4) | 1,593 (26.8) |
| Workload: usually | 18,305 (6.8) | 533 (9.0) |
| Workload: always | 17,839 (6.6) | 613 (10.3) |
| Ethnicity: White | 255,422 (94.3) | 4,487 (75.5) |
| Ethnicity: Asian/Asian British or Chinese | 6,261 (2.3) | 333 (5.6) |
| Ethnicity: Black/Black British | 4,946 (1.8) | 146 (2.5) |
| Ethnicity: Mixed | 1,900 (0.7) | 40 (0.7) |
| Ethnicity: Other | 2,442 (0.9) | 107 (1.8) |
| Education: degree | 103,270 (38.1) | 693 (11.7) |
| Education: A/AS level | 33,366 (12.3) | 207 (3.5) |
| Education: vocational/professional | 62,079 (22.9) | 504 (8.5) |
| Education: GCSE/O level | 33,088 (12.2) | 216 (3.6) |
| Education: CSE | 12,339 (4.6) | 90 (1.5) |
| Education: none listed | 26,829 (9.9) | 275 (4.6) |
| Smoking: never | 155,916 (57.5) | 2,884 (48.5) |
| Smoking: former | 86,262 (31.8) | 1,588 (26.7) |
| Smoking: current | 28,793 (10.6) | 730 (12.3) |
| Alcohol: daily/almost daily | 53,346 (19.7) | 982 (16.5) |
| Alcohol: 3–4 times/week | 66,986 (24.7) | 1,209 (20.3) |
| Alcohol: once/twice/week | 74,989 (27.7) | 1,557 (26.2) |
| Alcohol: 1–3 times/month | 32,062 (11.8) | 710 (11.9) |
| Alcohol: special occasions only | 27,152 (10.0) | 758 (12.7) |
| Alcohol: never | 16,436 (6.1) | 559 (9.4) |
| Assessment center: England | 239,434 (88.4) | 5,579 (93.8) |
| Assessment center: Scotland | 20,032 (7.4) | 237 (4.0) |
| Assessment center: Wales | 11,505 (4.2) | 130 (2.2) |

Values are mean (SD) or n (%). The second group lacks at least one adjustment covariate. NR is not zero. Variable-level and exposure-group missingness, outcome fractions and observed/completed distributions are provided in Table S13. Descriptive BMI was missing for 172 complete cases and 6 participants with incomplete adjustment data. These are descriptive comparisons, not tests of missing at random.

## Supplementary Table S11A. Exposure-group events and person-time: Eligible population

| **Outcome** | **Exposure category** | **N** | **Events** | **Person-years** | **Rate / 1,000 PY** |
| --- | --- | --- | --- | --- | --- |
| Disc disorders (M51) | Lower frequency / WHtR <0.5 | NR | 498 | 612,321.6 | 0.813 |
| Disc disorders (M51) | Higher frequency / WHtR <0.5 | 10,934 | 102 | 74,992.9 | 1.360 |
| Disc disorders (M51) | Lower frequency / WHtR ≥0.5 | NR | 1,210 | 1,037,702.5 | 1.166 |
| Disc disorders (M51) | Higher frequency / WHtR ≥0.5 | 26,356 | 343 | 181,387.6 | 1.891 |
| Radiculopathy subtype | Lower frequency / WHtR <0.5 | NR | 233 | 613,134.6 | 0.380 |
| Radiculopathy subtype | Higher frequency / WHtR <0.5 | 10,934 | 45 | 75,180.1 | 0.599 |
| Radiculopathy subtype | Lower frequency / WHtR ≥0.5 | NR | 558 | 1,039,798.4 | 0.537 |
| Radiculopathy subtype | Higher frequency / WHtR ≥0.5 | 26,356 | 154 | 181,989.8 | 0.846 |

First events and risk time are outcome-specific. These crude rates are not standardized risks. NR means a numerical cell was not released; eligible-population events are provided for all four categories without combining eligible and complete-case denominators.

## Supplementary Table S11B. Exposure-group events and person-time: Complete-case population

| **Outcome** | **Exposure category** | **N** | **Events** | **Person-years** | **Rate / 1,000 PY** |
| --- | --- | --- | --- | --- | --- |
| Disc disorders (M51) | Lower frequency / WHtR <0.5 | 87,376 | 480 | 600,735.9 | 0.799 |
| Disc disorders (M51) | Higher frequency / WHtR <0.5 | 10,666 | NR | 72,957.1 | NR |
| Disc disorders (M51) | Lower frequency / WHtR ≥0.5 | 147,451 | 1,163 | 1,011,472.0 | 1.150 |
| Disc disorders (M51) | Higher frequency / WHtR ≥0.5 | 25,478 | NR | 174,847.3 | NR |
| Radiculopathy subtype | Lower frequency / WHtR <0.5 | 87,376 | 224 | 601,526.9 | 0.372 |
| Radiculopathy subtype | Higher frequency / WHtR <0.5 | 10,666 | NR | 73,144.0 | NR |
| Radiculopathy subtype | Lower frequency / WHtR ≥0.5 | 147,451 | 534 | 1,013,485.3 | 0.527 |
| Radiculopathy subtype | Higher frequency / WHtR ≥0.5 | 25,478 | NR | 175,419.8 | NR |

First events and risk time are outcome-specific. These crude rates are not standardized risks. NR means a numerical cell was not released; eligible-population events are provided for all four categories without combining eligible and complete-case denominators.

## Supplementary Table S12A. Additional sensitivity analyses: Disc disorders (M51)

| **Sensitivity** | **Exposure contrast** | **Sensitivity N** | **Sensitivity events** | **Comparison model HR (95% CI)** | **Sensitivity HR (95% CI)** |
| --- | --- | --- | --- | --- | --- |
| Education strata | Higher frequency / WHtR <0.5 | 270,971 | 2,072 | 1.467 (1.178–1.826) | 1.466 (1.178–1.826) |
| Education strata | Lower frequency / WHtR ≥0.5 | 270,971 | 2,072 | 1.398 (1.251–1.562) | 1.398 (1.251–1.563) |
| Education strata | Higher frequency / WHtR ≥0.5 | 270,971 | 2,072 | 2.042 (1.751–2.380) | 2.041 (1.751–2.379) |
| Education strata | WHtR per 0.05 | 270,971 | 2,072 | 1.130 (1.098–1.163) | 1.130 (1.098–1.163) |
| Education strata | Higher vs lower frequency | 270,971 | 2,072 | 1.469 (1.312–1.645) | 1.468 (1.311–1.644) |
| England/Scotland | Higher frequency / WHtR <0.5 | 259,466 | 1,995 | 1.467 (1.178–1.826) | 1.434 (1.146–1.795) |
| England/Scotland | Lower frequency / WHtR ≥0.5 | 259,466 | 1,995 | 1.398 (1.251–1.562) | 1.393 (1.244–1.559) |
| England/Scotland | Higher frequency / WHtR ≥0.5 | 259,466 | 1,995 | 2.042 (1.751–2.380) | 2.035 (1.741–2.379) |
| England/Scotland | WHtR per 0.05 | 259,466 | 1,995 | 1.130 (1.098–1.163) | 1.130 (1.098–1.164) |
| England/Scotland | Higher vs lower frequency | 259,466 | 1,995 | 1.469 (1.312–1.645) | 1.461 (1.302–1.640) |
| Additional weekly hours | Higher frequency / WHtR <0.5 | 267,227 | 2,050 | 1.494 (1.200–1.861) | 1.496 (1.201–1.863) |
| Additional weekly hours | Lower frequency / WHtR ≥0.5 | 267,227 | 2,050 | 1.403 (1.255–1.569) | 1.402 (1.253–1.568) |
| Additional weekly hours | Higher frequency / WHtR ≥0.5 | 267,227 | 2,050 | 2.053 (1.759–2.395) | 2.049 (1.756–2.391) |

Categorical contrasts use lower frequency/WHtR <0.5 as reference. Continuous WHtR and binary frequency estimates belong to the continuous model. PH comparators use the original complete cases. England/Scotland comparators are the full original complete-case model and therefore differ in population; the country restriction is defined by assessment center. Weekly-hours comparisons use exactly the same valid-hours participants before/after adding one hours/10 term. All retain the 2015 cutoff. N and event counts refer to the sensitivity sample. The full-cohort comparator uses 270,971 participants (2,072 M51 or 951 subtype events); the work-hours comparator instead uses the identical 267,227-person valid-hours subset.

## Supplementary Table S12B. Additional sensitivity analyses: Radiculopathy subtype

| **Sensitivity** | **Exposure contrast** | **Sensitivity N** | **Sensitivity events** | **Comparison model HR (95% CI)** | **Sensitivity HR (95% CI)** |
| --- | --- | --- | --- | --- | --- |
| Sex strata | Higher frequency / WHtR <0.5 | 270,971 | 951 | 1.354 (0.975–1.881) | 1.355 (0.975–1.882) |
| Sex strata | Lower frequency / WHtR ≥0.5 | 270,971 | 951 | 1.411 (1.198–1.660) | 1.411 (1.199–1.661) |
| Sex strata | Higher frequency / WHtR ≥0.5 | 270,971 | 951 | 1.996 (1.591–2.504) | 1.994 (1.589–2.502) |
| Sex strata | WHtR per 0.05 | 270,971 | 951 | 1.138 (1.090–1.187) | 1.138 (1.090–1.187) |
| Sex strata | Higher vs lower frequency | 270,971 | 951 | 1.407 (1.189–1.665) | 1.406 (1.188–1.664) |
| England/Scotland | Higher frequency / WHtR <0.5 | 259,466 | 922 | 1.354 (0.975–1.881) | 1.328 (0.949–1.858) |
| England/Scotland | Lower frequency / WHtR ≥0.5 | 259,466 | 922 | 1.411 (1.198–1.660) | 1.400 (1.187–1.651) |
| England/Scotland | Higher frequency / WHtR ≥0.5 | 259,466 | 922 | 1.996 (1.591–2.504) | 2.020 (1.605–2.541) |
| England/Scotland | WHtR per 0.05 | 259,466 | 922 | 1.138 (1.090–1.187) | 1.142 (1.094–1.193) |
| England/Scotland | Higher vs lower frequency | 259,466 | 922 | 1.407 (1.189–1.665) | 1.421 (1.198–1.686) |
| Additional weekly hours | Higher frequency / WHtR <0.5 | 267,227 | 942 | 1.376 (0.990–1.912) | 1.377 (0.991–1.914) |
| Additional weekly hours | Lower frequency / WHtR ≥0.5 | 267,227 | 942 | 1.409 (1.196–1.660) | 1.408 (1.195–1.659) |
| Additional weekly hours | Higher frequency / WHtR ≥0.5 | 267,227 | 942 | 2.026 (1.614–2.543) | 2.023 (1.612–2.540) |

Categorical contrasts use lower frequency/WHtR <0.5 as reference. Continuous WHtR and binary frequency estimates belong to the continuous model. PH comparators use the original complete cases. England/Scotland comparators are the full original complete-case model and therefore differ in population; the country restriction is defined by assessment center. Weekly-hours comparisons use exactly the same valid-hours participants before/after adding one hours/10 term. All retain the 2015 cutoff. N and event counts refer to the sensitivity sample. The full-cohort comparator uses 270,971 participants (2,072 M51 or 951 subtype events); the work-hours comparator instead uses the identical 267,227-person valid-hours subset.

## Supplementary Table S13A. Adjustment-data missingness by exposure category

| **Variable** | **Eligible overall** | **Lower/<0.5** | **Higher/<0.5** | **Lower/≥0.5** | **Higher/≥0.5** |
| --- | --- | --- | --- | --- | --- |
| Ethnicity | 833 (0.30%) | NR | NR | NR | NR |
| Education | 3,961 (1.43%) | NR | NR | NR | NR |
| Townsend score | 385 (0.14%) | NR | 19 (0.17%) | NR | 35 (0.13%) |
| Smoking | 744 (0.27%) | NR | 31 (0.28%) | NR | 130 (0.49%) |
| Alcohol | 171 (0.06%) | NR | 12 (0.11%) | NR | 30 (0.11%) |
| Any adjustment variable | 5,946 (2.15%) | NR | 268 (2.45%) | NR | 878 (3.33%) |

Overall eligible N = 276,917. Higher-frequency/<0.5 N = 10,934; higher-frequency/≥0.5 N = 26,356. Some historical exposure-group counts and missingness cells remain NR. Age, sex and regional fields were complete within the eligible cohort. Missing exposures had been excluded at cohort construction and were not imputed.

## Observed outcome fractions by adjustment-data status

| **Population** | **Outcome** | **N** | **Events** | **Event fraction (%)** |
| --- | --- | --- | --- | --- |
| Complete adjustment data | Disc disorders (M51) | 270,971 | 2,072 | 0.765 |
| Complete adjustment data | Radiculopathy subtype | 270,971 | 951 | 0.351 |
| Missing adjustment data | Disc disorders (M51) | 5,946 | 81 | 1.362 |
| Missing adjustment data | Radiculopathy subtype | 5,946 | 39 | 0.656 |

Fractions are descriptive, not time-adjusted incidence rates and not evidence that missingness is ignorable.

## Supplementary Table S13B. Observed and completed covariate distributions

| **Variable** | **Category** | **Observed statistic** | **Mean across completions** | **Range across completions** |
| --- | --- | --- | --- | --- |
| Ethnicity | White | 94.141 | 94.128 | 94.123–94.132 |
| Ethnicity | Mixed | 0.703 | 0.704 | 0.702–0.705 |
| Ethnicity | Asian/Asian British or Chinese | 2.388 | 2.393 | 2.390–2.397 |
| Ethnicity | Black/Black British | 1.844 | 1.850 | 1.847–1.855 |
| Ethnicity | Other | 0.923 | 0.925 | 0.922–0.927 |
| Education | Degree | 38.088 | 37.911 | 37.884–37.934 |
| Education | A/AS | 12.300 | 12.257 | 12.241–12.266 |
| Education | Vocational/professional | 22.928 | 22.987 | 22.958–23.030 |
| Education | GCSE/O levels | 12.201 | 12.235 | 12.220–12.248 |
| Education | CSE | 4.553 | 4.615 | 4.601–4.635 |
| Education | None | 9.930 | 9.994 | 9.974–10.011 |
| Smoking | Never | 57.500 | 57.490 | 57.481–57.505 |
| Smoking | Former | 31.810 | 31.817 | 31.803–31.824 |
| Smoking | Current | 10.690 | 10.693 | 10.689–10.699 |
| Alcohol | Daily/almost daily | 19.631 | 19.628 | 19.624–19.632 |
| Alcohol | 3–4/week | 24.642 | 24.638 | 24.634–24.640 |
| Alcohol | 1–2/week | 27.659 | 27.657 | 27.652–27.660 |
| Alcohol | 1–3/month | 11.842 | 11.843 | 11.840–11.845 |
| Alcohol | Special occasions | 10.085 | 10.088 | 10.085–10.091 |
| Alcohol | Never | 6.141 | 6.146 | 6.143–6.148 |
| Townsend score | continuous | -1.330 | -1.330 | -1.330–-1.330 |

Categorical entries are percentages among observed nonmissing values or, after imputation, all 276,917 participants. Townsend entries are means. The same participants appear in each of 20 completed datasets; denominators are not multiplied by 20. These are observed-variable versus completed-data distributions, not an independent validation sample. Standard deviations and complete numerical summaries accompany this table as CSV.

## Supplementary Table S14. Multiple-imputation pooled exposure effects and FMI

| **Outcome** | **Exposure category** | **HR (95% CI)** | **FMI** | **Degrees of freedom** |
| --- | --- | --- | --- | --- |
| Disc disorders (M51) | Higher frequency / WHtR <0.5 | 1.44 (1.16–1.78) | 0.0012 | 2126.4 |
| Disc disorders (M51) | Lower frequency / WHtR ≥0.5 | 1.40 (1.25–1.56) | 0.0011 | 2126.7 |
| Disc disorders (M51) | Higher frequency / WHtR ≥0.5 | 2.03 (1.75–2.36) | 0.0019 | 2124.8 |
| Radiculopathy subtype | Higher frequency / WHtR <0.5 | 1.33 (0.96–1.84) | 0.0025 | 963.6 |
| Radiculopathy subtype | Lower frequency / WHtR ≥0.5 | 1.40 (1.20–1.65) | 0.0022 | 963.9 |
| Radiculopathy subtype | Higher frequency / WHtR ≥0.5 | 1.95 (1.56–2.44) | 0.0035 | 962.6 |

Reference: lower frequency/WHtR <0.5. N = 276,917; M51/subtype events = 2,153/990. Twenty completed datasets; Rubin variance with Barnard–Rubin degrees of freedom. FMI is the fraction of missing information, not the missing-data proportion. Covariate-specific results for all 48 coefficients, final predictor inclusion, and observed/completed summaries are in the companion CSVs. Final saved chains retained disease/death indicators and all four cumulative-hazard auxiliaries.

## Supplementary Table S15. Ethnicity specification and exposure estimates

| **Outcome** | **Specification** | **Higher/<0.5** | **Lower/≥0.5** | **Higher/≥0.5** |
| --- | --- | --- | --- | --- |
| Disc disorders (M51) | Original six-category coefficients | 1.467 (1.178–1.826) | 1.393 (1.246–1.556) | 2.034 (1.745–2.371) |
| Disc disorders (M51) | Pooled five-category coefficients | 1.467 (1.178–1.826) | 1.398 (1.251–1.562) | 2.042 (1.751–2.380) |
| Disc disorders (M51) | Original six-category strata | 1.467 (1.178–1.826) | 1.394 (1.247–1.558) | 2.034 (1.745–2.372) |
| Radiculopathy subtype | Original six-category coefficients | 1.354 (0.975–1.881) | 1.406 (1.194–1.654) | 1.989 (1.585–2.495) |
| Radiculopathy subtype | Pooled five-category coefficients | 1.354 (0.975–1.881) | 1.411 (1.198–1.660) | 1.996 (1.591–2.504) |
| Radiculopathy subtype | Original six-category strata | 1.355 (0.975–1.883) | 1.406 (1.195–1.655) | 1.991 (1.587–2.498) |

All use the same 270,971 complete cases and 2015 cutoff. Values are HRs (95% CIs), relative to lower frequency/WHtR <0.5. The original six-category coefficient model was numerically unstable: Chinese-category HR was approximately 8.66×10⁻⁷ for M51 (SE of log HR 403.84) and 8.33×10⁻⁷ for the subtype (SE 595.75), with unbounded upper CIs. Finite exposure estimates do not establish stability of those original models. Full original coefficients and diagnostics are supplied in CSV. Pooling and the stratified sensitivity were selected with knowledge of preliminary results and not as blinded, preregistered decisions.

## Supplementary Table S16. Repeated employment and heavy-work responses within follow-up

| **Assessment instance** | **Observed response/availability** | **Participants (released interval)** |
| --- | --- | --- |
| 1 | assessment attendance | 11200-11299 |
| 1 | work hours | 7400-7499 |
| 1 | workload frequency | 7500-7599 |
| 1 | shift work | 7500-7599 |
| 1 | night shift | 1000-1099 |
| 2 | assessment attendance | 4500-4599 |
| 2 | work hours | 2800-2899 |
| 2 | workload frequency | 2800-2899 |
| 2 | shift work | 2800-2899 |
| 2 | night shift | 400-499 |
| 1 | unknown invalid | 10-19 |
| 1 | paid not retired | 7150-7159 |
| 1 | paid and retired | 400-409 |
| 1 | retired not paid | 3260-3269 |
| 1 | other known not paid | 380-389 |
| 1 | Work frequency low → low | 6610-6619 |
| 1 | Work frequency low → high | 230-239 |
| 1 | Work frequency high → low | 210-219 |
| 1 | Work frequency high → high | 490-499 |
| 2 | unknown invalid | 40-49 |
| 2 | paid not retired | 2710-2719 |
| 2 | paid and retired | 120-129 |
| 2 | retired not paid | 1500-1509 |
| 2 | other known not paid | 150-159 |
| 2 | Work frequency low → low | 2460-2469 |
| 2 | Work frequency low → high | 90-99 |
| 2 | Work frequency high → low | 110-119 |
| 2 | Work frequency high → high | 150-159 |

Instance 1 is the first repeat assessment; instance 2 is the first imaging assessment. Intervals are released counts, not estimates with confidence limits; no midpoint was substituted. People can attend both visits, so counts cannot be summed as independent participants. Employment allows multiple responses, including paid work and retirement together. Descriptive visits can occur after disease events and do not identify exact retirement dates. Instance 3 falls outside the historical window and contributes no observations here.

Supplementary Figure S1. Unadjusted cumulative incidence of intervertebral disc disorders. Aalen–Johansen estimates account for competing death. Lines join an exported regular time grid; exact individual event times are not displayed. Bands are pointwise 95% confidence intervals. These unadjusted estimates concern the eligible population, not the standardized complete-case target population.

Supplementary Figure S2. Unadjusted cumulative incidence of disc-related radiculopathy. Aalen–Johansen estimates account for competing death. Lines join an exported regular time grid and bands represent pointwise 95% confidence intervals. An earlier non-radiculopathy M51 diagnosis does not end radiculopathy follow-up.

Supplementary Figure S3. Waist-to-height ratio and intervertebral disc disorders. The curve and band show adjusted hazard ratios and pointwise 95% confidence intervals from a four-knot restricted cubic spline, with WHtR 0.5 as reference. The displayed range is the 1st–99th percentile range. Adjustment includes occupational workload and the primary covariates. Nonlinearity P = 0.263; this analysis does not identify a disease threshold.

Supplementary Figure S4. Waist-to-height ratio and disc-related radiculopathy. Restricted cubic spline specification and adjustment are the same as in Supplementary Figure S3; WHtR 0.5 is the reference. The displayed range is restricted to the 1st–99th percentiles. Nonlinearity P = 0.308.

Supplementary Figure S5. Original four-level occupational workload analysis. Points and bars are adjusted hazard ratios and 95% confidence intervals relative to never/rarely undertaking heavy physical work. Models adjust for continuous WHtR and the primary covariates. The categories represent reported frequency, not equally spaced biomechanical doses. Radiculopathy point estimates are not strictly monotonic.
